# Supplementary figures and images for: A novel recombinant PHB production platform in filamentous cyanobacteria avoiding nitrogen starvation while preserving cell viability
Source: Microb Cell Fact. 2025 Feb 20;24:43. doi: 10.1186/s12934-025-02650-y (PMC11844001; doi:10.1186/s12934-025-02650-y)

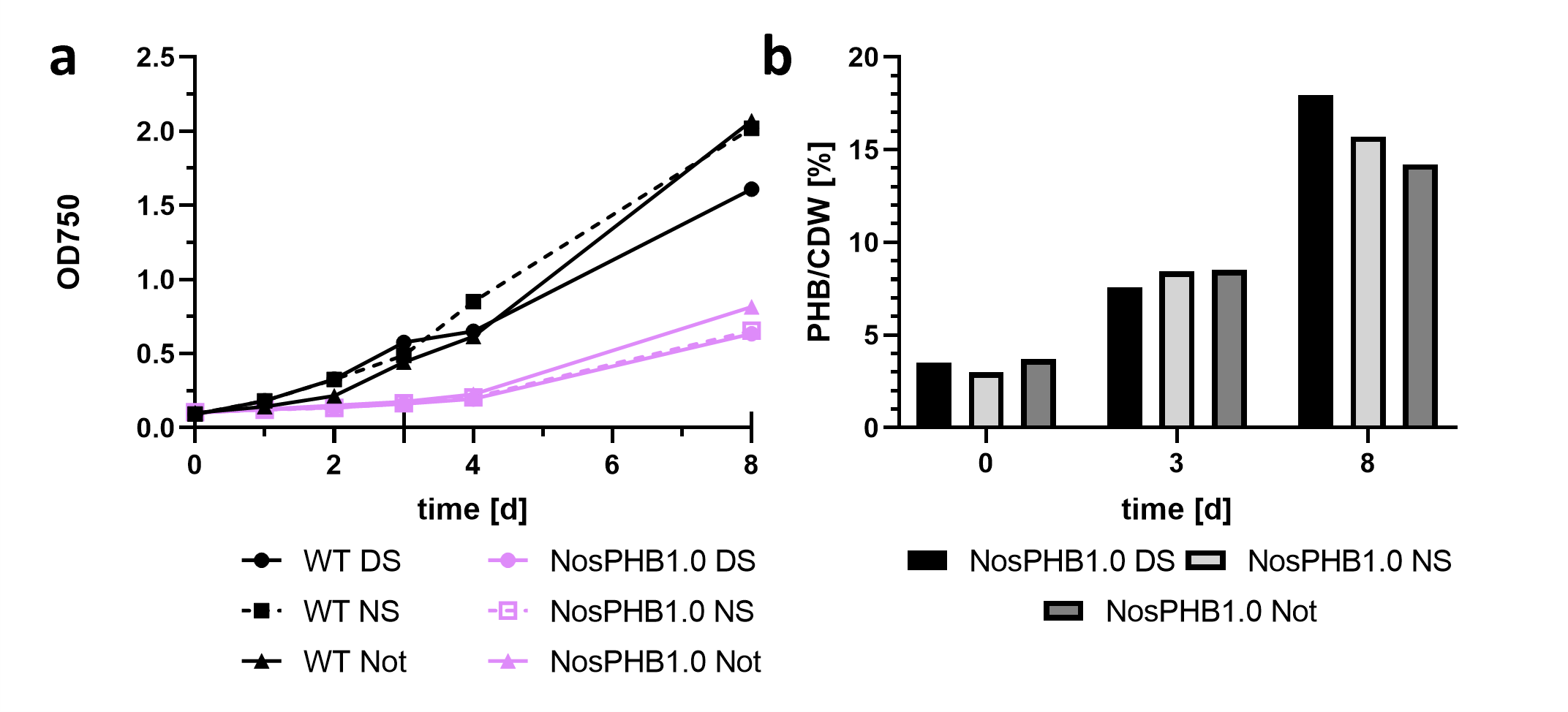

Supplement: Supplementary file 4 — Supplementary Material 4 [file 12934_2025_2650_MOESM4_ESM.tif]

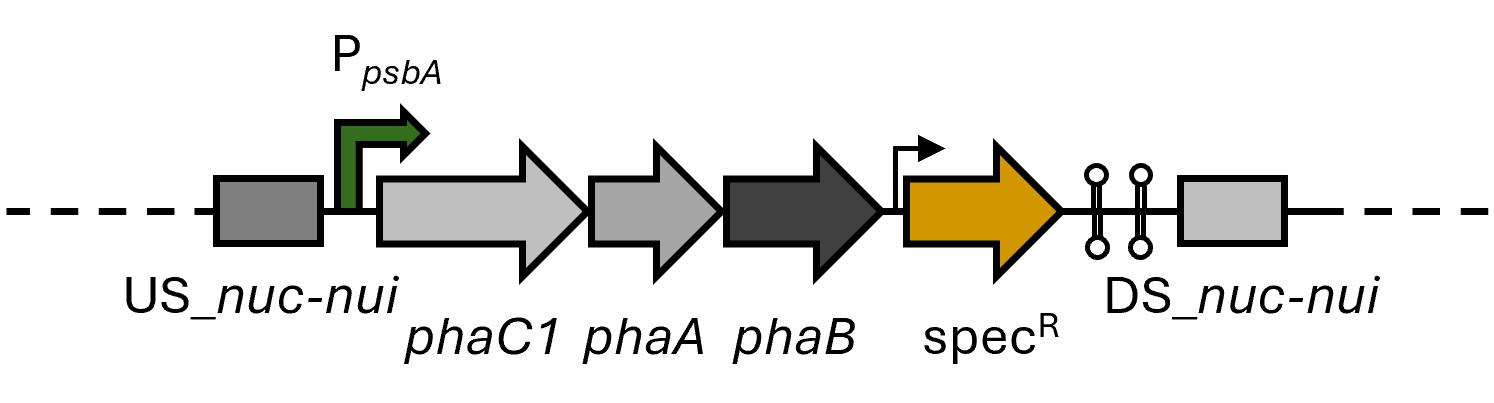

Supplement: Supplementary file 5 — Supplementary Material 5 [file 12934_2025_2650_MOESM5_ESM.tif]

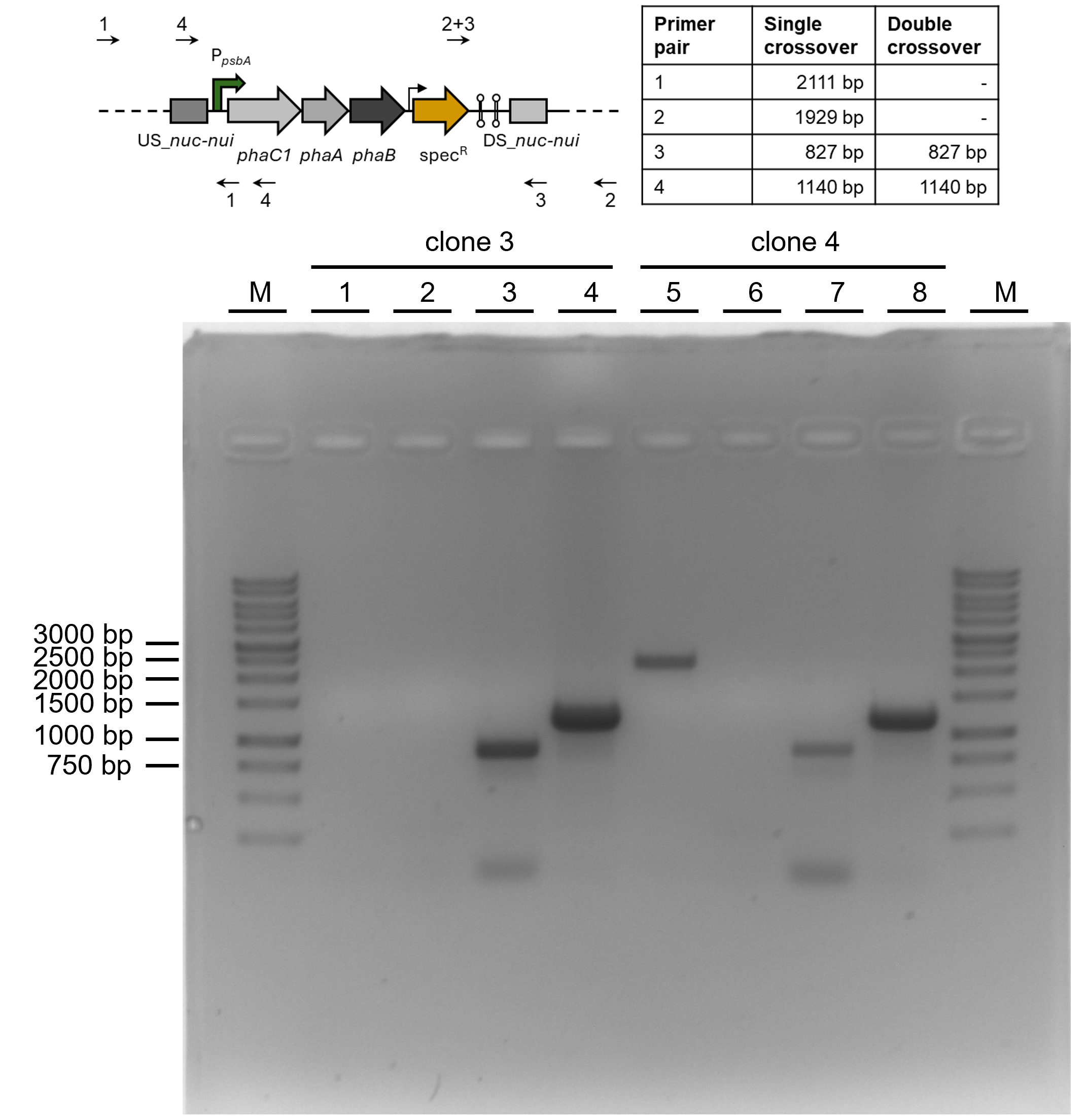

Supplement: Supplementary file 7 — Supplementary Material 7 [file 12934_2025_2650_MOESM7_ESM.tif]

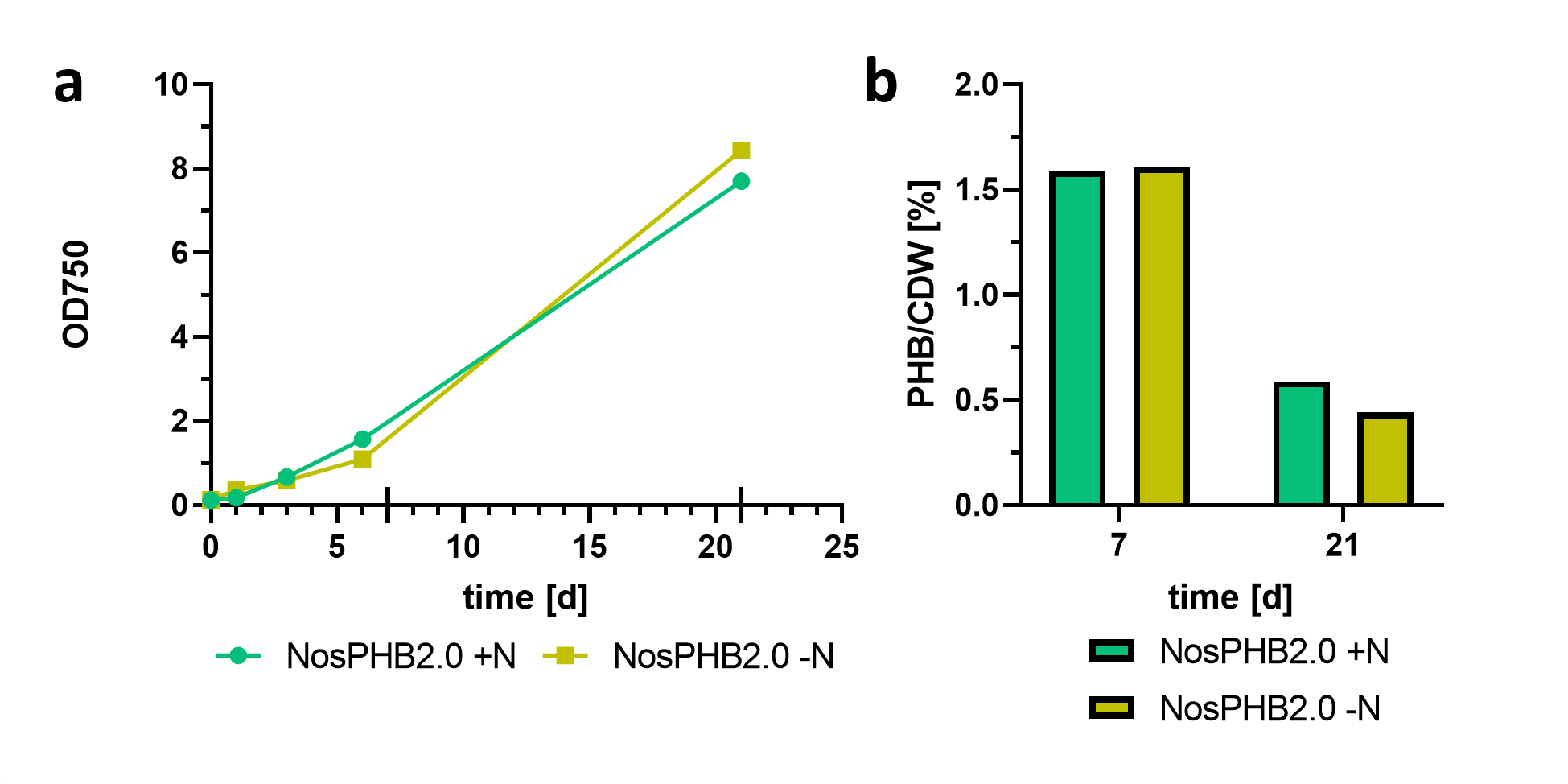

Supplement: Supplementary file 8 — Supplementary Material 8 [file 12934_2025_2650_MOESM8_ESM.tif]

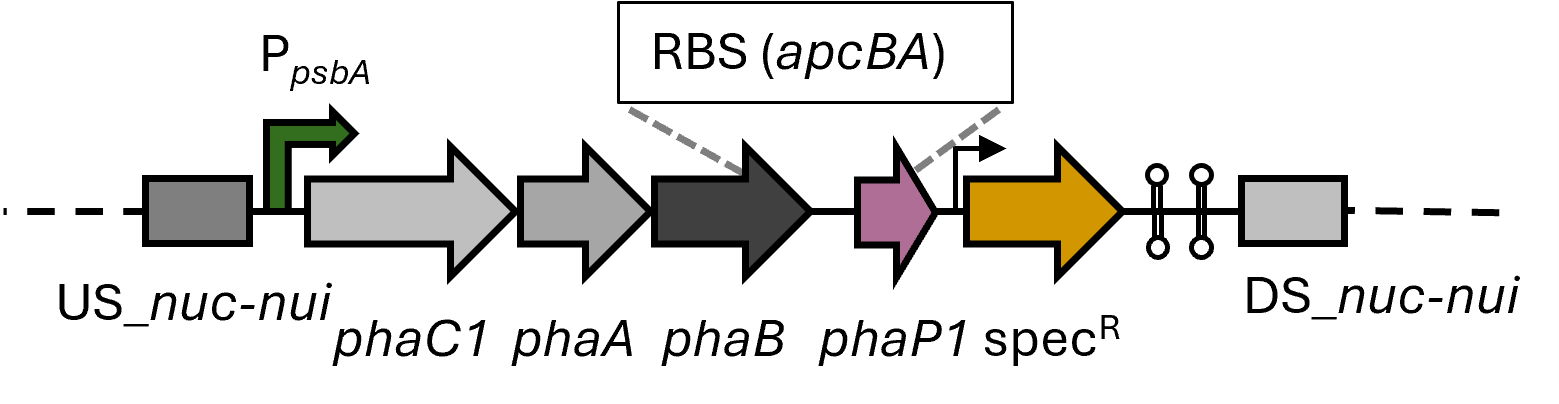

Supplement: Supplementary file 9 — Supplementary Material 9 [file 12934_2025_2650_MOESM9_ESM.tif]

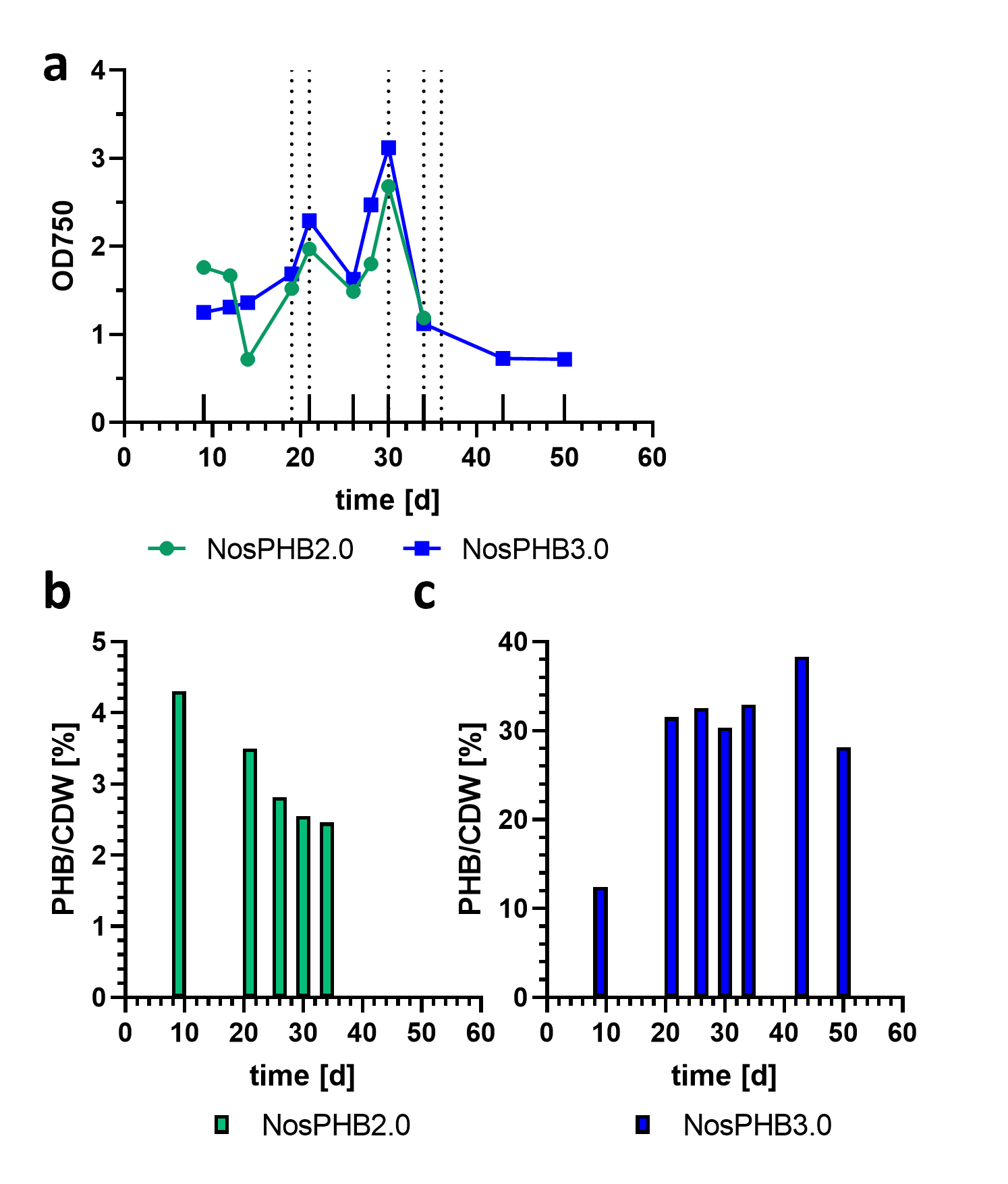

Supplement: Supplementary file 11 — Supplementary Material 11 [file 12934_2025_2650_MOESM11_ESM.tif]

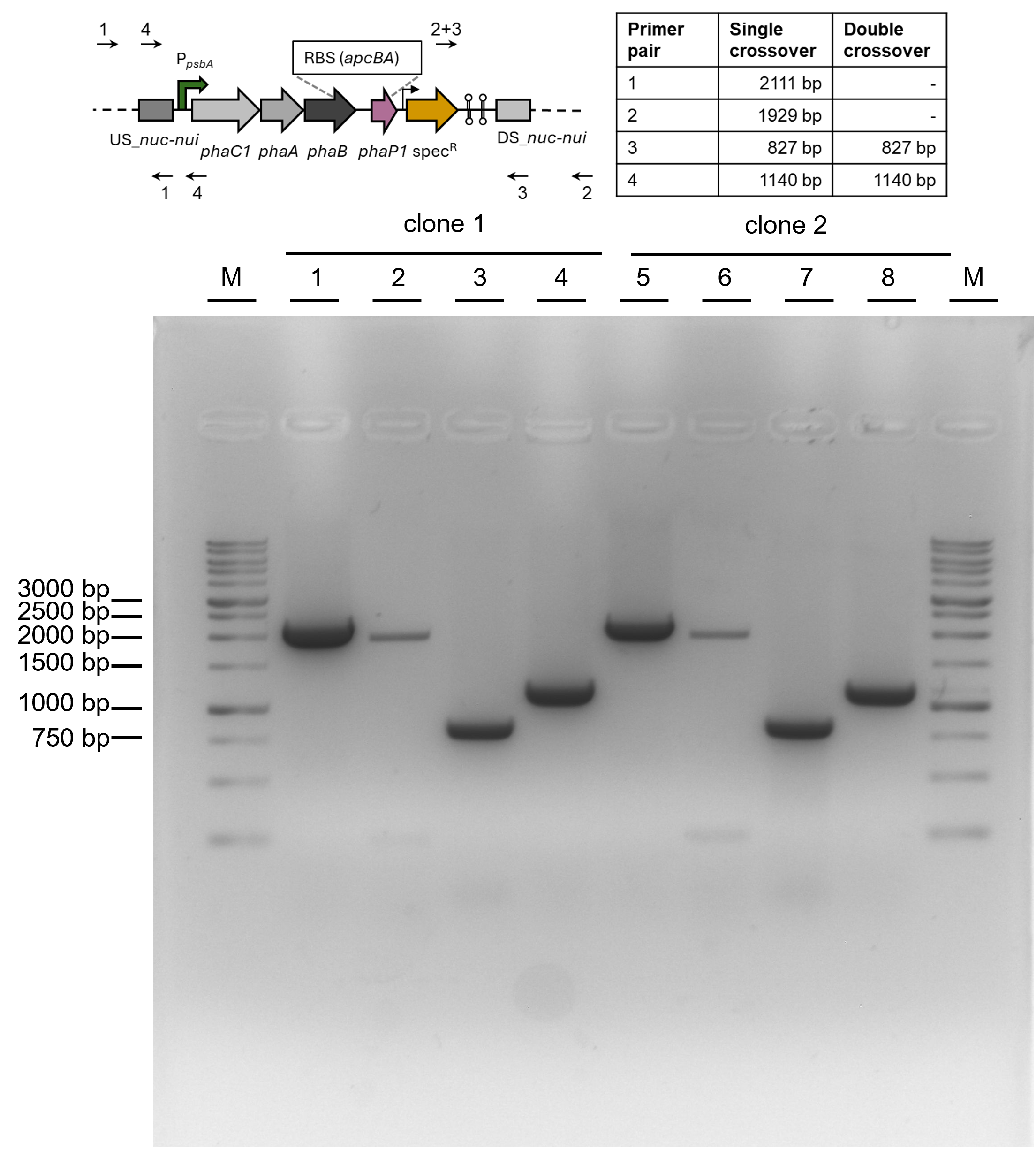

Supplement: Supplementary file 12 — Supplementary Material 12 [file 12934_2025_2650_MOESM12_ESM.tif]

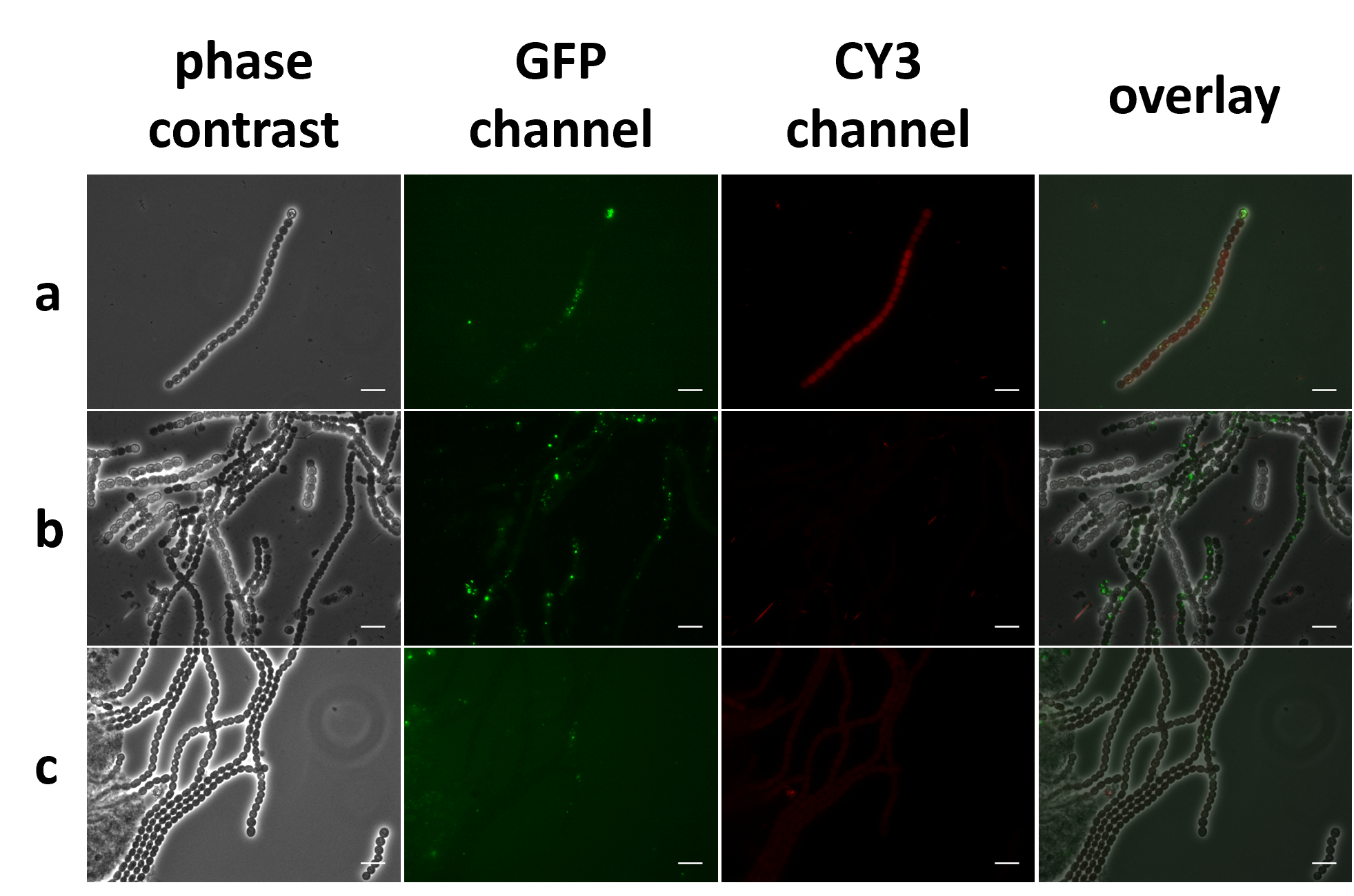

Supplement: Supplementary file 13 — Supplementary Material 13 [file 12934_2025_2650_MOESM13_ESM.tif]

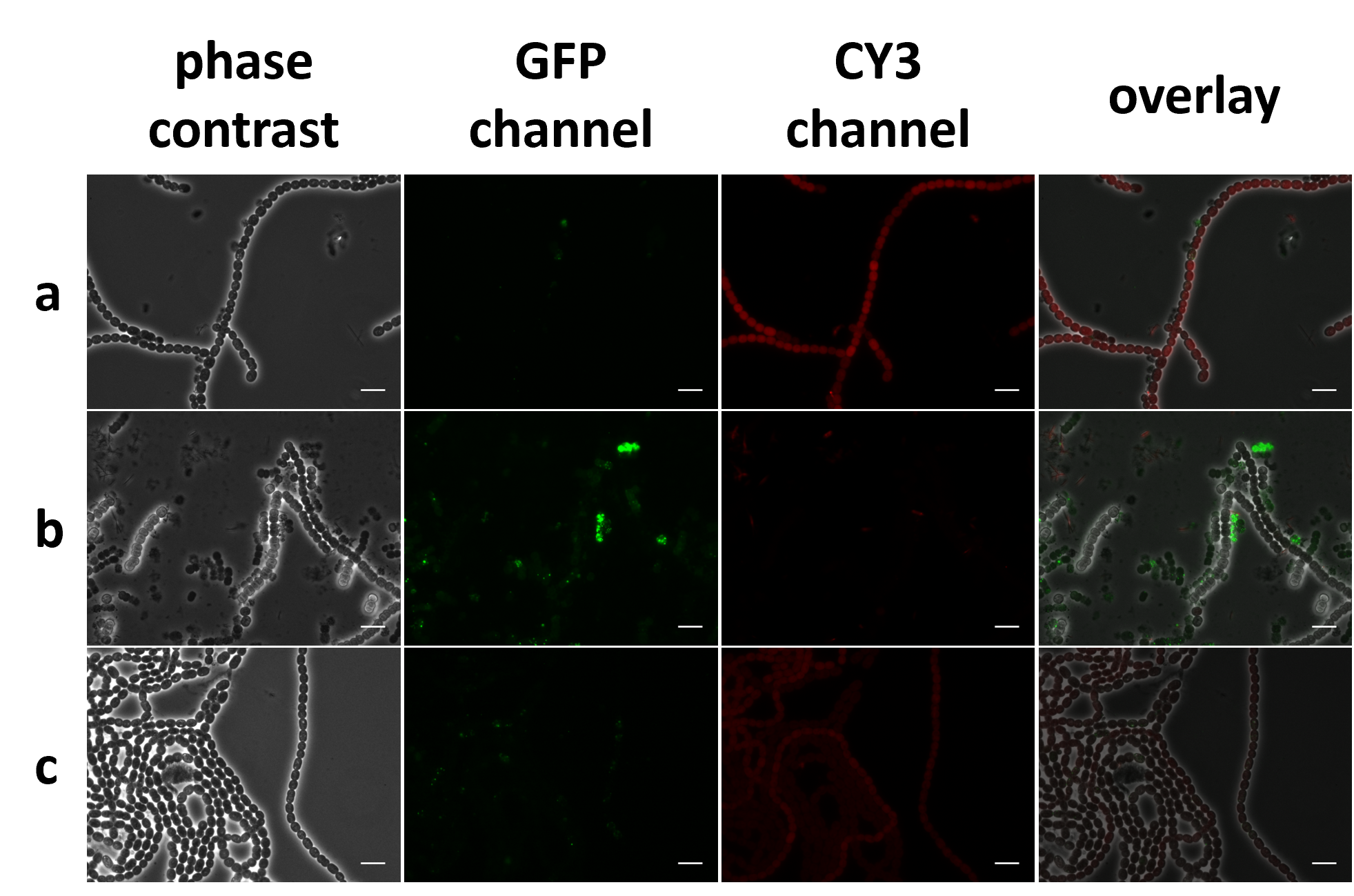

Supplement: Supplementary file 14 — Supplementary Material 14 [file 12934_2025_2650_MOESM14_ESM.tif]

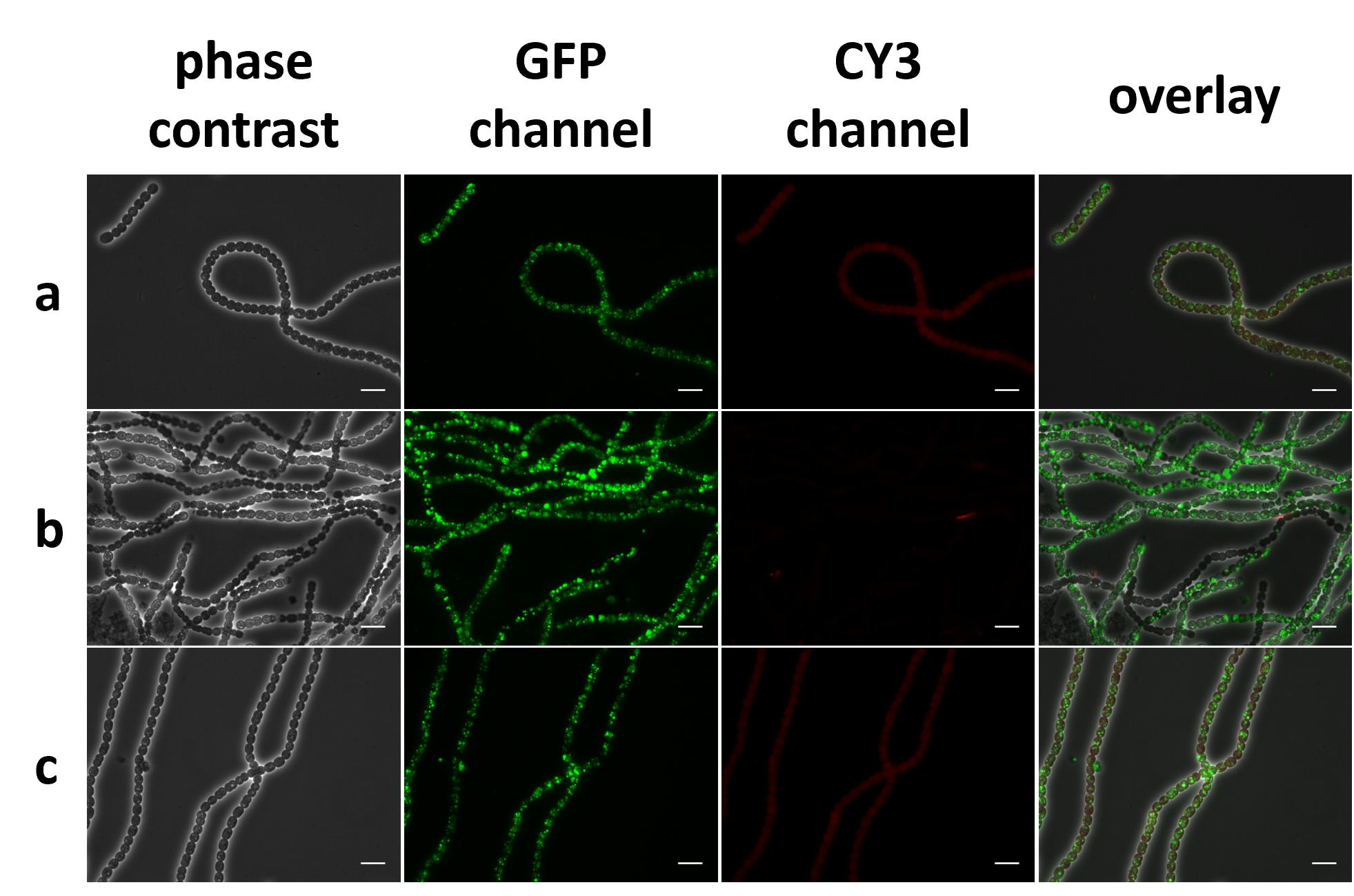

Supplement: Supplementary file 15 — Supplementary Material 15 [file 12934_2025_2650_MOESM15_ESM.tif]

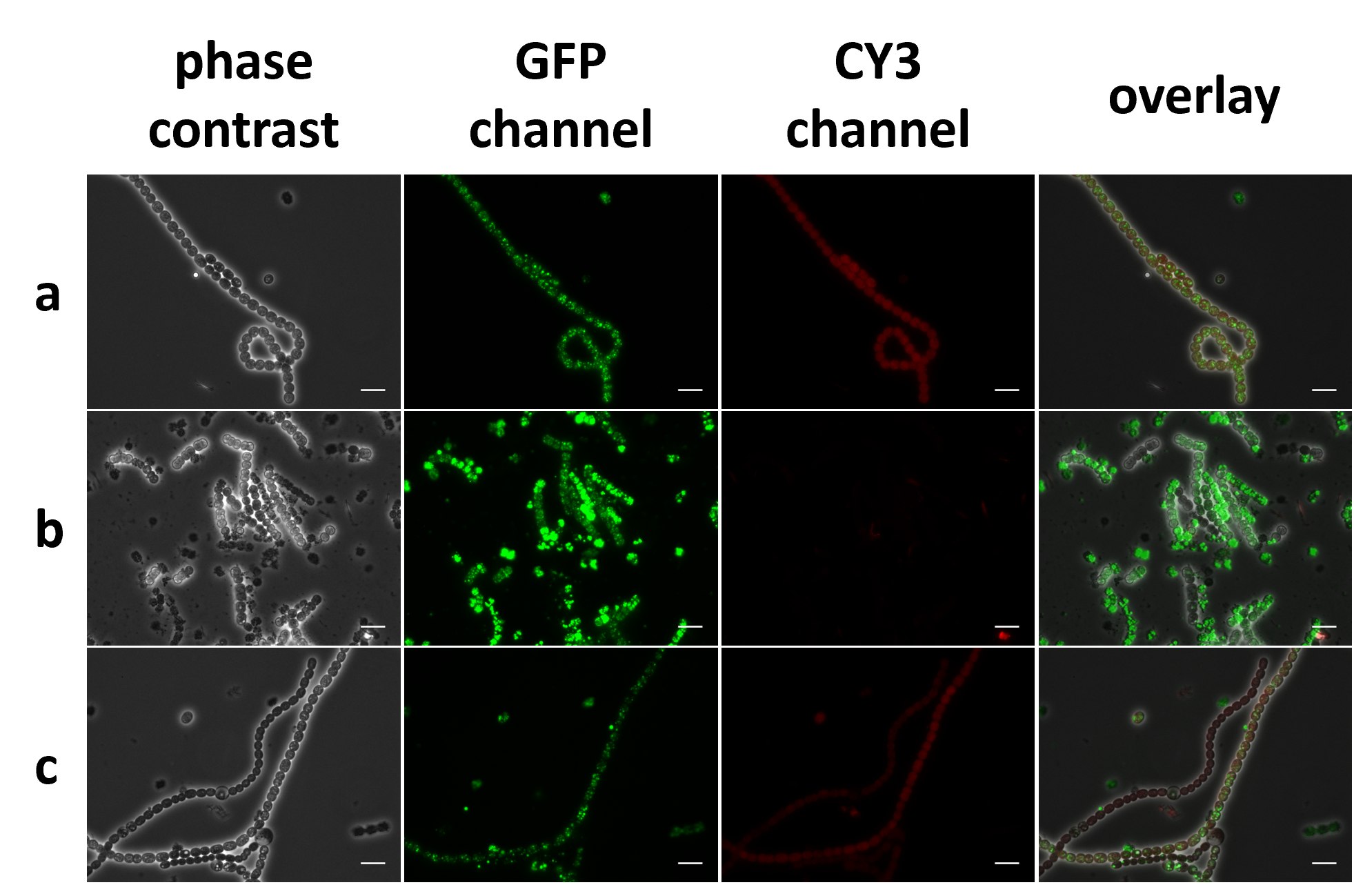

Supplement: Supplementary file 16 — Supplementary Material 16 [file 12934_2025_2650_MOESM16_ESM.tif]
