# Supplementary material for: A novel recombinant PHB production platform in filamentous cyanobacteria avoiding nitrogen starvation while preserving cell viability
Source: Microb Cell Fact. 2025 Feb 20;24:43. doi: 10.1186/s12934-025-02650-y (PMC11844001; doi:10.1186/s12934-025-02650-y)
Supplement: Supplementary file 17 — Supplementary Material 17 [file 12934_2025_2650_MOESM17_ESM.docx]

A novel recombinant PHB production platform in filamentous cyanobacteria avoiding nitrogen starvation while preserving cell viability

Phillipp Fink^1^, Claudia Menzel^1^, Jong-Hee Kwon^2,3^, Karl Forchhammer^1, *^

^1^Organismic Interactions Department, Tübingen University, Auf der Morgenstelle 28, 72076 Tübingen, Germany

^2^Division of Applied Life Sciences (BK21), Gyeongsang National University, Jinju 52828, Republic of Korea
^3^Department of Food Science & Technology and Institute of Agriculture & Life Science, Gyeongsang National University, Jinju 52828, Republic of Korea

*Corresponding author’s email: Karl.Forchhammer@uni-tuebingen.de

**Supplementary information:**

Additional file 1: Table S 1: Strains used and created in this study

| Name | Genotype | Reference |
| --- | --- | --- |
| *E. coli* NEB10β | Δ(*ara-leu*)7697 *araD139 fhuA* Δ*lacX74 galK16 galE15 e14^-^ϕ80dlacZΔM15 recA1 relA1 endA1 nupG rpsL* (Sm^R^) *rph spoT1* Δ(*mrr*-*hsdRMS*-*mcrBC),* Sm^R^ | NEB |
| *E. coli* Stellar cells | F- *endA1 supE44 thi-1 recA1 relA1 gyrA96 phoA Φ80dlacZΔM15* Δ(lacZYA‑argF) U169 Δ(*mrr‑hsdRMS*-*mcrBC*), Δ*mcrA*, λ- | TAKARA |
| *E. coli* J53/RP4 | RP4, Ap^R^, Km^R^, Tc^R^ | Datta 1971 (1) |
| *E. coli* HB101 | F– *thi*-1 *hsd*S20(r_B_^–^, m_B_^–^) *sup*E44 *rec*A13 *ara*-14 *leu*B6 *pro*A2 *lac*Y1 *gal*K2 *rps*L20 (Sm^R^) *xyl*-5 *mtl*-1, Sm^R^ | Promega |
| *E. coli* HB101/ pRL528 | HB101+ pRL528, Cm^R^, Sm^R^ | Elhai 1997 (2) |
| *E. coli* HB101/ pRL528/pRL1049-P_psbA_-*phaCAB* | HB101+ pRL528 + pRL1049-P_psbA_-*phaCAB,* Cm^R^, Sm^R^/Sp^R^ | This study |
| *E. coli* HB101/ pRL528/pPF08 | HB101 + pPF08, Cm^R^, Sm^R^/Sp^R^, Em^R^ | This study |
| *E. coli* HB101/ pRL528/pPF10 | HB101 + pPF10, Cm^R^, Sm^R^/Sp^R^, Em^R^ | This study |
| *Nostoc* sp. PCC7120 | Wild-type strain | Pasteur culture collection PCC |
| NosPHB1.0 | *Nostoc* sp. PCC7120 with replicative plasmid pRL1049-P_psbA_-*phaCAB*; Sm^R^/Sp^R^ | This study |
| NosPHB2.0 | *Nostoc* sp. PCC7120 with integrated PHB operon (P_psbA_-*phaCAB*) inside neutral site (*nucA*-*nuiA* region), Sm^R^/Sp^R^ | This study |
| NosPHB3.0 | *Nostoc* sp. PCC7120 with integrated PHB operon (P_psbA_-*phaCAB*-*apcBA*(*phaP*)) inside neutral site (*nucA*-*nuiA* region), Sm^R^/Sp^R^ | This study |
| Abbreviations: Ap: Ampicillin, Cm: Chloramphenicol, Sm: Streptomycin, Sp: Spectinomycin, Km: Kanamycin, Tc: Tetracycline | | |

Additional file 2: Table S 2: Primer used in this study

| Name | Primer | Description |
| --- | --- | --- |
| pPF08_fragment 1 fw | acattgcagttgagaacccagaagctgctgGCAATGGCAACAACGTTGCGCAAACTATTA | Gibson primer to create DNA fragment 1 for pPF08 |
| pPF08_fragment 1 rev | cagatccttcccacaaaaaaacctcaaatgGATCTAGATATCGAATTTCTGCCATTCATCCG | Gibson primer to create DNA fragment 1 for pPF08 |
| pPF08_fragment 2 fw | GATGAATGGCAGAAATTCGATATCTAGATCcatttgaggtttttttgtgggaaggatctg | Gibson primer to create DNA fragment 2 for pPF08 |
| pPF08_fragment 2 rev | GTCAACCAATATTCATTGAGATCCTCTAGAacacctgataattacctgatggtcaaaaat | Gibson primer to create DNA fragment 2 for pPF08 |
| pPF08_fragment 3 fw | atttttgaccatcaggtaattatcaggtgtTCTAGAGGATCTCAATGAATATTGGTTGACAC | Gibson primer to create DNA fragment 3 for pPF08 |
| pPF08_fragment 3 rev | ctgggaaatcccagtggtgcaacgccaacaGAGTTTGTAGAAACGCAAAAAGGCCATCCG | Gibson primer to create DNA fragment 3 for pPF08 |
| pPF08_fragment 4 fw | CGGATGGCCTTTTTGCGTTTCTACAAACTCtgttggcgttgcaccactgggatttcccag | Gibson primer to create DNA fragment 4 for pPF08 |
| pPF08_fragment 4 rev | TAATAGTTTGCGCAACGTTGTTGCCATTGCcagcagcttctgggttctcaactgcaatgt | Gibson primer to create DNA fragment 4 for pPF08 |
| pPF10_fragment 1 fw | GCCACGGCAAAGAAGACGACGGCTGCCTGAGAGCTCTTGACCGAACGCAGCGGTGGTAAC | Gibson primer to create DNA fragment 1 for pPF10 |
| pPF10_fragment 1 rev | ttccagtttcagcagggaaacagctgcaccTCAGCCCATATGCAGGCCGCCGTTGAGCGA | Gibson primer to create DNA fragment 1 for pPF10 |
| verification primer 1fw | CCTGGTGTCCCTGTTGATAC | Primer for double crossover verification in recombinant *Nostoc* strain |
| verification primer 1rev | CATGTGGCTGGATCGAATG | Primer for double crossover verification in recombinant *Nostoc* strain |
| verification primer 4fw | GTTAAGCTGCCAAGCTACCC | Primer for double crossover verification in recombinant *Nostoc* strain |
| verification primer 4rev | AGCACGTTGATCTTGTCCTG | Primer for double crossover verification in recombinant *Nostoc* strain |
| verification primer 2+3fw | ACATAGCGTTGCCTTGGTAG | Primer for double crossover verification in recombinant *Nostoc* strain |
| verification primer 2rev | AATGATACCGCGAGACCCAC | Primer for double crossover verification in recombinant *Nostoc* strain |
| verification primer 3rev | TCCCAAGTGCCACCATTAAC | Primer for double crossover verification in recombinant *Nostoc* strain |

Additional file 3: Table S 3: Plasmids used in this study

| Name | Characteristics | Reference or Source |
| --- | --- | --- |
| RP4 | R+, met, pro, Tra+ IncP; Ap^R^, Km^R^, Tc^R^ | Datta 1971 (1) |
| pRL528 | Helper plasmid for mobilization used in  triparental mating:  Mob_ColK_, M.AvaI, M.Eco47II, Cm^R^ | Elhai 1997 (2) |
| pRL271 | Vector for triparental conjugation and subsequent *sacB*-mediated positive selection of double crossover mutants in *Nostoc* sp. PCC7120, Cm^R^, Em^R^ | Cai and Wolk 1990 (3) |
| pRL1049 | Self-replicating plasmid, Sm^R^/Sp^R^ | Black 1994 (4) |
| pRL1049-P_psbA_-*phaCAB* | pRL1049 harboring PHB operon P*_psbA_*-*phaCAB*, Sm^R^/Sp^R^ | Kindly provided by Jörg Scholl |
| pPF08 | pRL271 with homologous regions of *nucA*-*nuiA* and expanded PHB operon P_psbA_-*phaCAB* with spectinomycin/streptomycin resistance cassette *aad1* and t1t2 terminator, Sm^R^/Sp^R^, Cm^R^, Em^R^ | This study |
| pPF10 | pRL271 with homologous regions of *nucA*-*nuiA* and expanded PHB operon P_psbA_-*phaCAB* with insertion of intergenic region of *abcBA* and *phaP* and spectinomycin/streptomycin resistance cassette *aad1* and t1t2 terminator, Sm^R^/Sp^R^, Cm^R^, Em^R^ | This study |
| Abbreviations: Ap: Ampicillin, Cm: Chloramphenicol, Em: Erythromycin, Sm: Streptomycin, Sp: Spectinomycin | | |


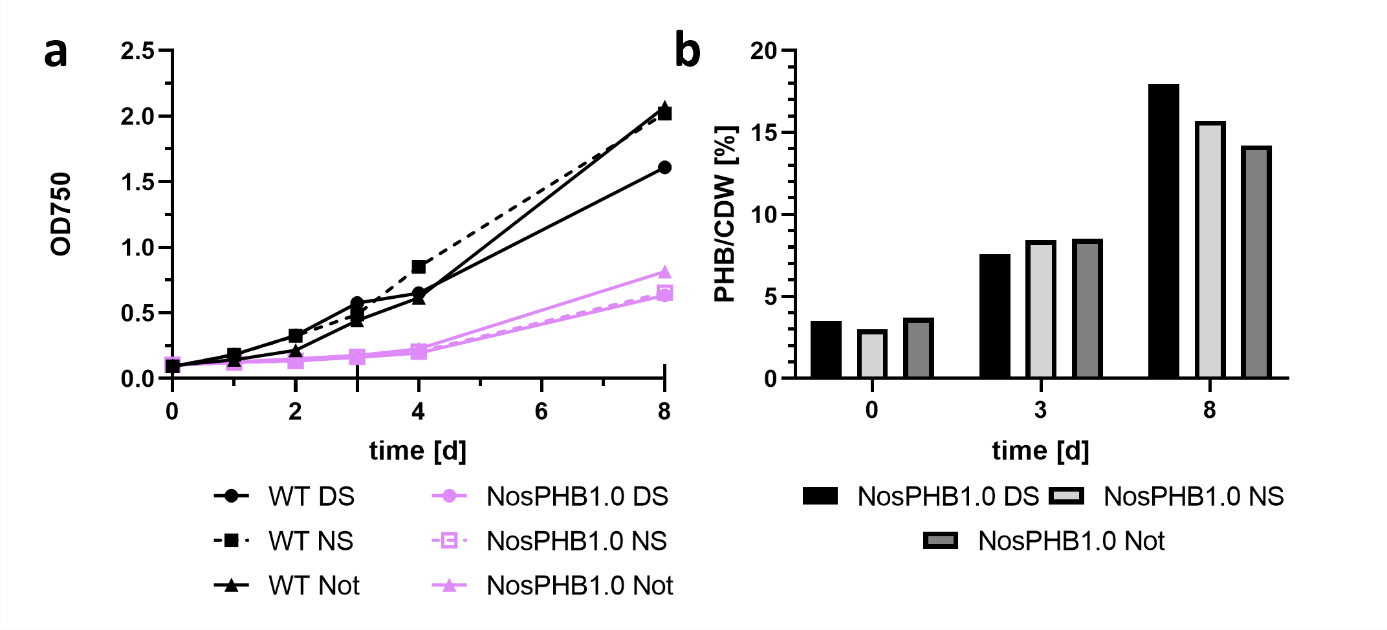


Additional file 4 Fig. S 1: Growth curve and PHB content of NosPHB1.0 measured under different conditions grown in BG11 medium. **(a)** Growth curve of wild-type strain *Nostoc* sp. PCC7120 (black) and NosPHB1.0 (violet) under various growth conditions, DS: 20 µmol m^-2^s^-1^, 120 rpm; NS: 40 µmol m^-2^s^-1^, 120 rpm; Not: 20 µmol m^-2^s^-1^, no shaking, recorded by measuring the OD_750_. Longer ticks on the x-axis indicate samples taken for PHB quantification. Each point represents one biological replica recorded by measuring the OD_750_. Longer ticks on the x-axis indicate samples taken for PHB quantification. Each point represents one biological replicate**(b)** PHB content of NosPHB1.0 after 0, 3 and 8 days of cultivation under various growth conditions. Each data set represents one biological sample.


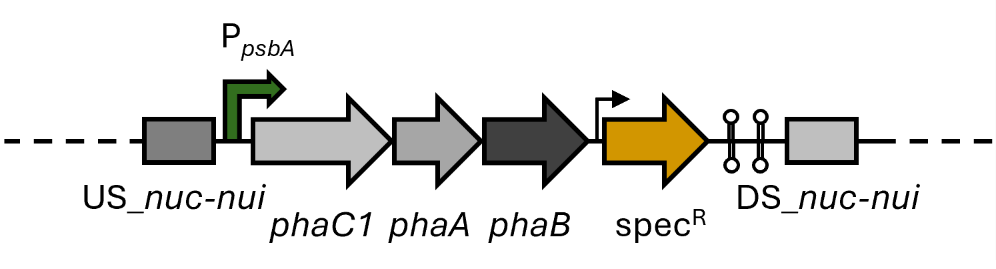


Additional file 5 Fig. S 2: Schematic image of modified PHB in pPF08 for creating recombinant *Nostoc* strain NosPHB2.0; US_*nuc-nui:* Upstream region (1000 bp) of neutral site *nuc-nui*; P*_psbA_*: constitutive promotor; *phaC1*; PHB polymerase; *phaA*: β-ketothiolase*; phaB:* acetoacetyl-CoA reductase; spec^R^: spectinomycin resistance gene *aad1*, an adenylytransferase; DS_*nuc-nui:* Downstream region (1000 bp) of neutral site *nuc-nui.*

Additional file 6 Table S 4: nucleotide sequence of modified PHB operon in pPF08

| >US_*nuc-nui*: catttgaggtttttttgtgggaaggatctgcacctcctgttacacatgaaatagttttgcagcaaacaggtcacggacaagatgcgccttttaaagtggtagacattgacagcttttttagcagagccactactccccaagactggtatgaggatgaagaaaatgctgtagttgctaaatttcaaaaactgctagaggtaataaaatcgaacttaaaaaacccgcaggtgtatcgactgggtgaggtagaacttgatgtttatgttattggtgaaactccagcaggaaatttagctggtatttctactaaagttgtggaaacttgacctatttactaattatcaactttactctcaatacttgtttgaatattgggggaaacattagacaaaaaatcataacccgtcaaactttctaattcatcaacactgactttataagccctccagtcattatttaattctgggtcgttgggaatatttactgcgataacgcgagtattagcagtaataccttcaagccctgagcctgggctatctagtacgacaacaatcttccaagtggatttgggaactgtcaccttacctttgaggggtttgccaagactaccattaggcccggcaacaatgtaaagctctttaccctgactgactaattctcgacaataatcttctaaatttccccacgtatttctattgttatcgggtgtttggggcatcatgtttgtcatcaggaaagtagccgcattatcttctgttgtcttggtgcggtctgctgaaggtgcaatatgcccccggtcataaccactcccagagtacatagaaggagtcactcgcacccaacccgcaggcaatgttttgtctgggcggaagttatcttgacgctctgcgttccctagccatgaggagttaagctgccaagctacccagttagcagttcccttgctgttgttgtaggagagtgcatattgatttttgaccatcaggtaattatcaggtgt |
| --- |
| >constitutive promotor P*_psbA_*:  TCTAGAGGATCTCAATGAATATTGGTTGACACGGGCGTATAAGACATGTTATACTGTTGAATAACAAGGACGGATCTGATCAAGAGACAGGATGAGGATC |
| PHB operon *phaC1AB:*  ATGGCGACCGGCAAAGGCGCGGCAGCTTCCACGCAGGAAGGCAAGTCCCAACCATTCAAGGTCACGCCGGGGCCATTCGATCCAGCCACATGGCTGGAATGGTCCCGCCAGTGGCAGGGCACTGAAGGCAACGGCCACGCGGCCGCGTCCGGCATTCCGGGCCTGGATGCGCTGGCAGGCGTCAAGATCGCGCCGGCGCAGCTGGGTGATATCCAGCAGCGCTACATGAAGGACTTCTCAGCGCTGTGGCAGGCCATGGCCGAGGGCAAGGCCGAGGCCACCGGTCCGCTGCACGACCGGCGCTTCGCCGGCGACGCATGGCGCACCAACCTCCCATATCGCTTCGCTGCCGCGTTCTACCTGCTCAATGCGCGCGCCTTGACCGAGCTGGCCGATGCCGTCGAGGCCGATGCCAAGACCCGCCAGCGCATCCGCTTCGCGATCTCGCAATGGGTCGATGCGATGTCGCCCGCCAACTTCCTTGCCACCAATCCCGAGGCGCAGCGCCTGCTGATCGAGTCGGGCGGCGAATCGCTGCGTGCCGGCGTGCGCAACATGATGGAAGACCTGACACGCGGCAAGATCTCGCAGACCGACGAGAGCGCGTTTGAGGTCGGCCGCAATGTCGCGGTGACCGAAGGCGCCGTGGTCTTCGAGAACGAGTACTTCCAGCTGTTGCAGTACAAGCCGCTGACCGACAAGGTGCACGCGCGCCCGCTGCTGATGGTGCCGCCGTGCATCAACAAGTACTACATCCTGGACCTGCAGCCGGAGAGCTCGCTGGTGCGCCATGTGGTGGAGCAGGGACATACGGTGTTTCTGGTGTCGTGGCGCAATCCGGACGCCAGCATGGCCGGCAGCACCTGGGACGACTACATCGAGCACGCGGCCATCCGCGCCATCGAAGTCGCGCGCGACATCAGCGGCCAGGACAAGATCAACGTGCTCGGCTTCTGCGTGGGCGGCACCATTGTCTCGACCGCGCTGGCGGTGCTGGCCGCGCGCGGCGAGCACCCGGCCGCCAGCGTCACGCTGCTGACCACGCTGCTGGACTTTGCCGACACGGGCATCCTCGACGTCTTTGTCGACGAGGGCCATGTGCAGTTGCGCGAGGCCACGCTGGGCGGCGGCGCCGGCGCGCCGTGCGCGCTGCTGCGCGGCCTTGAGCTGGCCAATACCTTCTCGTTCTTGCGCCCGAACGACCTGGTGTGGAACTACGTGGTCGACAACTACCTGAAGGGCAACACGCCGGTGCCGTTCGACCTGCTGTTCTGGAACGGCGACGCCACCAACCTGCCGGGGCCGTGGTACTGCTGGTACCTGCGCCACACCTACCTGCAGAACGAGCTCAAGGTACCGGGCAAGCTGACCGTGTGCGGCGTGCCGGTGGACCTGGCCAGCATCGACGTGCCGACCTATATCTACGGCTCGCGCGAAGACCATATCGTGCCGTGGACCGCGGCCTATGCCTCGACCGCGCTGCTGGCGAACAAGCTGCGCTTCGTGCTGGGTGCGTCGGGCCATATCGCCGGTGTGATCAACCCGCCGGCCAAGAACAAGCGCAGCCACTGGACTAACGATGCGCTGCCGGAGTCGCCGCAGCAATGGCTGGCCGGCGCCATCGAGCATCACGGCAGCTGGTGGCCGGACTGGACCGCATGGCTGGCCGGGCAGGCCGGCGCGAAACGCGCCGCGCCCGCCAACTATGGCAATGCGCGCTATCGCGCAATCGAACCCGCGCCTGGGCGATACGTCAAAGCCAAGGCATGACGCTTGCATGAGTGCCGGCGTGCGTCATGCACGGCGCCGGCAGGCCTGCAGGTTCCCTCCCGTTTCCATTGAAAGGACTACACAATGACTGACGTTGTCATCGTATCCGCCGCCCGCACCGCGGTCGGCAAGTTTGGCGGCTCGCTGGCCAAGATCCCGGCACCGGAACTGGGTGCCGTGGTCATCAAGGCCGCGCTGGAGCGCGCCGGCGTCAAGCCGGAGCAGGTGAGCGAAGTCATCATGGGCCAGGTGCTGACCGCCGGTTCGGGCCAGAACCCCGCACGCCAGGCCGCGATCAAGGCCGGCCTGCCGGCGATGGTGCCGGCCATGACCATCAACAAGGTGTGCGGCTCGGGCCTGAAGGCCGTGATGCTGGCCGCCAACGCGATCATGGCGGGCGACGCCGAGATCGTGGTGGCCGGCGGCCAGGAAAACATGAGCGCCGCCCCGCACGTGCTGCCGGGCTCGCGCGATGGTTTCCGCATGGGCGATGCCAAGCTGGTCGACACCATGATCGTCGACGGCCTGTGGGACGTGTACAACCAGTACCACATGGGCATCACCGCCGAGAACGTGGCCAAGGAATACGGCATCACACGCGAGGCGCAGGATGAGTTCGCCGTCGGCTCGCAGAACAAGGCCGAAGCCGCGCAGAAGGCCGGCAAGTTTGACGAAGAGATCGTCCCGGTGCTGATCCCGCAGCGCAAGGGCGACCCGGTGGCCTTCAAGACCGACGAGTTCGTGCGCCAGGGCGCCACGCTGGACAGCATGTCCGGCCTCAAGCCCGCCTTCGACAAGGCCGGCACGGTGACCGCGGCCAACGCCTCGGGCCTGAACGACGGCGCCGCCGCGGTGGTGGTGATGTCGGCGGCCAAGGCCAAGGAACTGGGCCTGACCCCGCTGGCCACGATCAAGAGCTATGCCAACGCCGGTGTCGATCCCAAGGTGATGGGCATGGGCCCGGTGCCGGCCTCCAAGCGCGCCCTGTCGCGCGCCGAGTGGACCCCGCAAGACCTGGACCTGATGGAGATCAACGAGGCCTTTGCCGCGCAGGCGCTGGCGGTGCACCAGCAGATGGGCTGGGACACCTCCAAGGTCAATGTGAACGGCGGCGCCATCGCCATCGGCCACCCGATCGGCGCGTCGGGCTGCCGTATCCTGGTGACGCTGCTGCACGAGATGAAGCGCCGTGACGCGAAGAAGGGCCTGGCCTCGCTGTGCATCGGCGGCGGCATGGGCGTGGCGCTGGCAGTCGAGCGCAAATAAGGAAGGGGTTTTCCGGGGCCGCGCGCGGTTGGCGCGGACCCGGCGACGATAACGAAGCCAATCAAGGAGTGGACATGACTCAGCGCATTGCGTATGTGACCGGCGGCATGGGTGGTATCGGAACCGCCATTTGCCAGCGGCTGGCCAAGGATGGCTTTCGTGTGGTGGCCGGTTGCGGCCCCAACTCGCCGCGCCGCGAAAAGTGGCTGGAGCAGCAGAAGGCCCTGGGCTTCGATTTCATTGCCTCGGAAGGCAATGTGGCTGACTGGGACTCGACCAAGACCGCATTCGACAAGGTCAAGTCCGAGGTCGGCGAGGTTGATGTGCTGATCAACAACGCCGGTATCACCCGCGACGTGGTGTTCCGCAAGATGACCCGCGCCGACTGGGATGCGGTGATCGACACCAACCTGACCTCGCTGTTCAACGTCACCAAGCAGGTGATCGACGGCATGGCCGACCGTGGCTGGGGCCGCATCGTCAACATCTCGTCGGTGAACGGGCAGAAGGGCCAGTTCGGCCAGACCAACTACTCCACCGCCAAGGCCGGCCTGCATGGCTTCACCATGGCACTGGCGCAGGAAGTGGCGACCAAGGGCGTGACCGTCAACACGGTCTCTCCGGGCTATATCGCCACCGACATGGTCAAGGCGATCCGCCAGGACGTGCTCGACAAGATCGTCGCGACGATCCCGGTCAAGCGCCTGGGCCTGCCGGAAGAGATCGCCTCGATCTGCGCCTGGTTGTCGTCGGAGGAGTCCGGTTTCTCGACCGGCGCCGACTTCTCGCTCAACGGCGGCCTGCATATGGGCTGA |
| >*aad1*:  GAGCTCTTGACCGAACGCAGCGGTGGTAACGGCGCAGTGGCGGTTTTCATGGCTTGTTATGACTGTTTTTTTGGGGTACAGTCTATGCCTCGGGCATCCAAGCAGCAAGCGCGTTACGCCGTGGGTCGATGTTTGATGTTATGGAGCAGCAACGATGTTACGCAGCAGGGCAGTCGCCCTAAAACAAAGTTAAACATCATGAGGGAAGCGGTGATCGCCGAAGTATCGACTCAACTATCAGAGGTAGTTGGCGTCATCGAGCGCCATCTCGAACCGACGTTGCTGGCCGTACATTTGTACGGCTCCGCAGTGGATGGCGGCCTGAAGCCACACAGTGATATTGATTTGCTGGTTACGGTGACCGTAAGGCTTGATGAAACAACGCGGCGAGCTTTGATCAACGACCTTTTGGAAACTTCGGCTTCCCCTGGAGAGAGCGAGATTCTCCGCGCTGTAGAAGTCACCATTGTTGTGCACGACGACATCATTCCGTGGCGTTATCCAGCTAAGCGCGAACTGCAATTTGGAGAATGGCAGCGCAATGACATTCTTGCAGGTATCTTCGAGCCAGCCACGATCGACATTGATCTGGCTATCTTGCTGACAAAAGCAAGAGAACATAGCGTTGCCTTGGTAGGTCCAGCGGCGGAGGAACTCTTTGATCCGGTTCCTGAACAGGATCTATTTGAGGCGCTAAATGAAACCTTAACGCTATGGAACTCGCCGCCCGACTGGGCTGGCGATGAGCGAAATGTAGTGCTTACGTTGTCCCGCATTTGGTACAGCGCAGTAACCGGCAAAATCGCGCCGAAGGATGTCGCTGCCGACTGGGCAATGGAGCGCCTGCCGGCCCAGTATCAGCCCGTCATACTTGAAGCTAGACAGGCTTATCTTGGACAAGAAGAAGATCGCTTGGCCTCGCGCGCAGATCAGTTGGAAGAATTTGTCCACTACGTGAAAGGCGAGATCACCAAGGTAGTCGGCAAATAA |
| >t_1_t_2_ terminator:  GCAGAAGCGGTCTGATAAAACAGAATTTGCCTGGCGGCAGTAGCGCGGTGGTCCCACCTGACCCCATGCCGAACTCAGAAGTGAAACGCCGTAGCGCCGATGGTAGTGTGGGGTCTCCCCATGCGAGAGTAGGGAACTGCCAGGCATCAAATAAAACGAAAGGCTCAGTCGAAAGACTGGGCCTTTCGTTTTATCTGTTGTTTGTCGGTGAACGCTCTCCTGAGTAGGACAAATCCGCCGGGAGCGGATTTGAACGTTGCGAAGCAACGGCCCGGAGGGTGGCGGGCAGGACGCCCGCCATAAACTGCCAGGCATCAAATTAAGCAGAAGGCCATCCTGACGGATGGCCTTTTTGCGTTTCTACAAACTC |
| >DS_nuc-nui:  tgttggcgttgcaccactgggatttcccagcagtaaatgcacgctgattgatggggaaagttcagttaatggtggcacttgggattggacaggcgaacacccgacaatcaacgccaccaacgccgctacacccaattttccacaaattcccataaattgacgcacctttagagatttatttcacttcaacacttgtgattgtatttcctgactttcactcagctaggcttcaaattttcaagtctcctgcccgatcaaccctaatttttcctctaaaaaaattagggttgctgacgatcagctacgctcttgcgttacgccatcgctctttcggtgtagcgtaggtcatcgcttacttttcctctctgcgagaggcgggagcgaacgcttgaatttggcggaagggctaaaccaaactcaattgcttcccgtattcctgctccaaacactcccccttgagtctaaagaaacacacacaccacctatcacccaccacccgttcaataccatgcggctgcttgttgtcaaaagcaatgcaatcgccctcctgcgttcatctcagtgtgccatatagtaagaaatagtaagaaaaattgctactttagagtaaaaataatgtattactatgtctaaagtataaaaaatcagtgttgccctgaccccagaaatggtagttttggttcgtgatgctgttgagtcaggagaatatgctagtagcagtgaggtaattcgtgaggcactgcgcgaatggaggcaaaaacggttacttcaattgcaaaatattgaggaactgcgccgtctttggcatgaaggaatggaaagtggtactggacgctttacagatatagaagccatcaagcaagaagcacgctctcgcttaggtcaaacaattcaaaaggatactgaactcaagtgggacgtttaattcgcacagctaaagcagaagaagacttaattgaaatatggatgtacattgcagttgagaacccagaagctgctg |


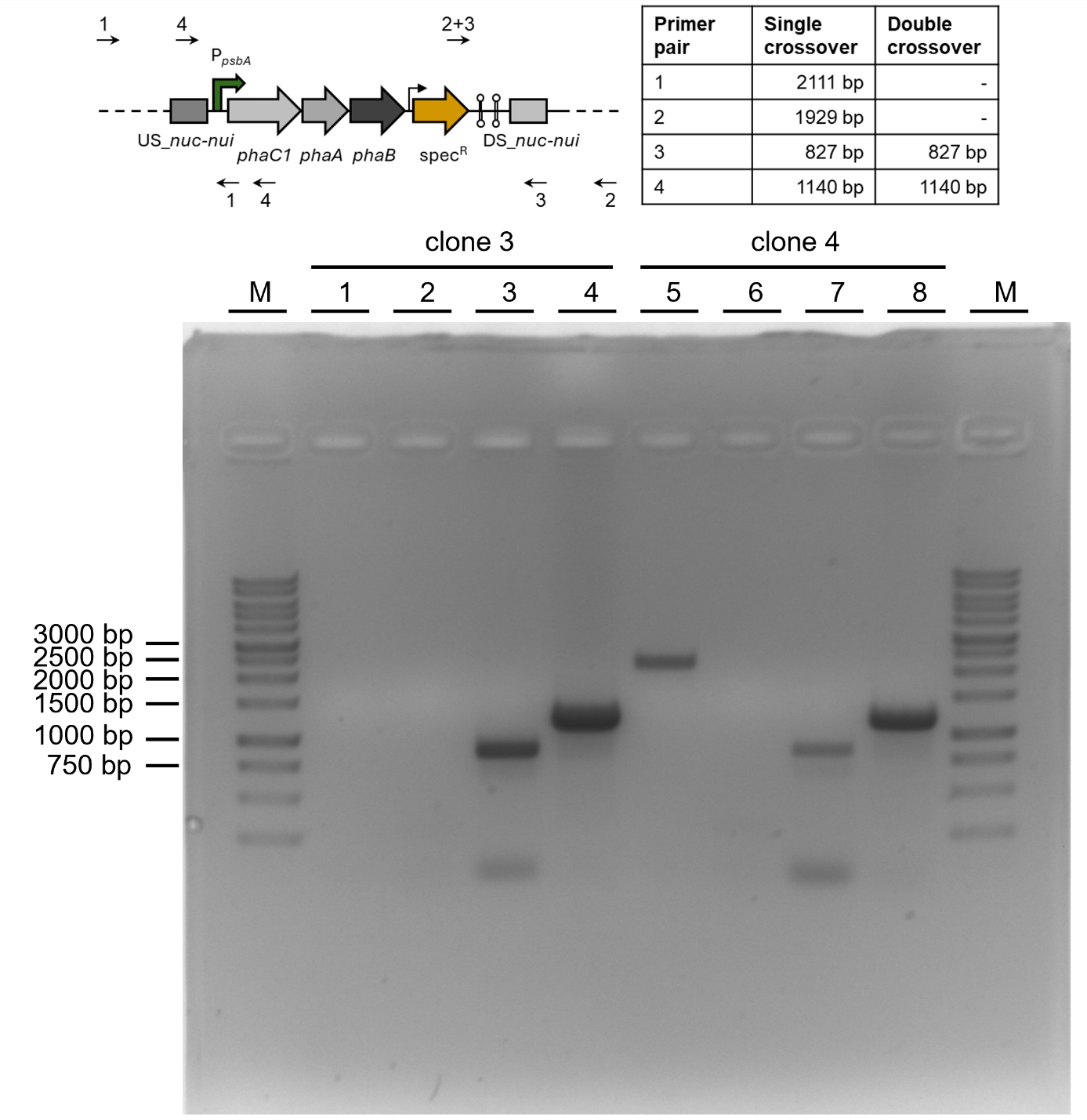


Additional file 7: Fig. S 3: Verification of successful genome integration of PHB operon in recombinant Nostoc strain NosPHB2.0; primer pairs 1-4 were chosen to verify successful double cross over event in recombinant *Nostoc* strain M: marker; line 1-4; clone 3 with primer pair 1-4 respectively; line 5-8; clone 4 with primer pair 1-4 respectively. Double crossover was achieved in clone 3.


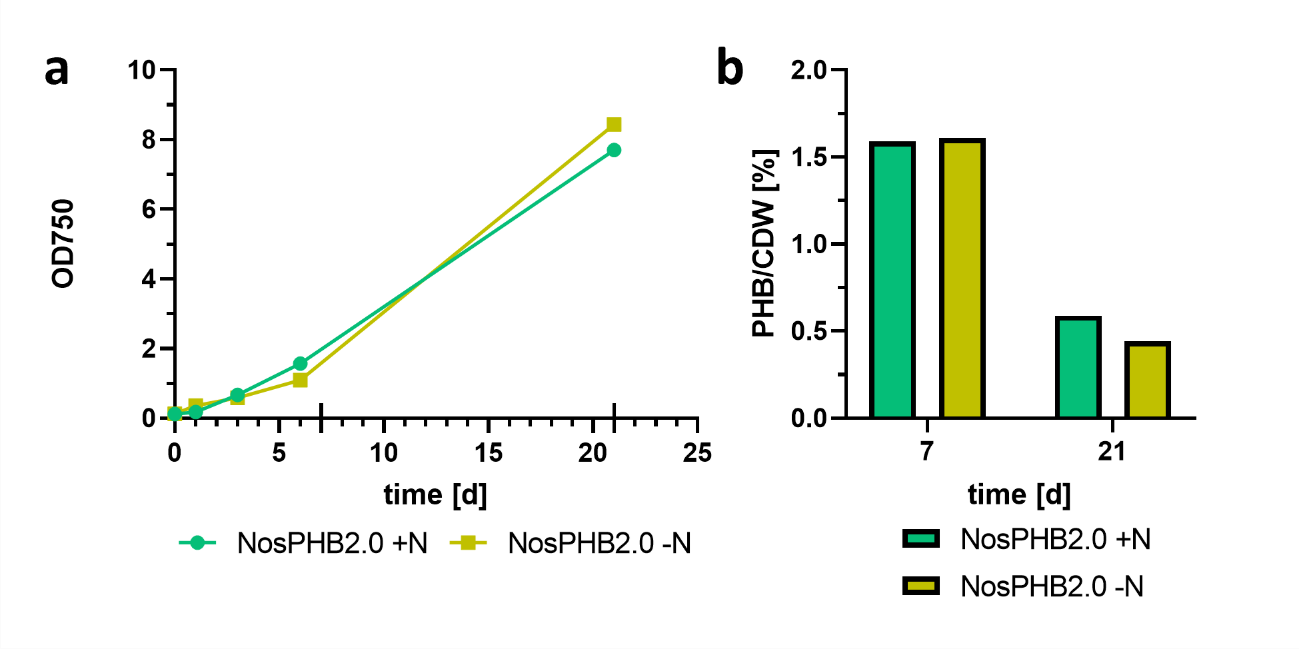


Additional file 8: Fig. S 4: Growth experiment and PHB content of NosPHB2.0 **(a)** Growth curve of NosPHB2.0 in BG11 (+N, green) and BG11_0_ (-N, yellow), Growth was recorded by measuring of the strain’s OD_750_, longer tick on x-axis indicates sample taken for PHB quantification. Each point represents one biological experiment **(b)** PHB content of NosPHB2.0 after 7 and 21 days of continuous growth condition in BG11 (+N, green) and BG11_0_ (-N, yellow). Each data set represents one biological sample.


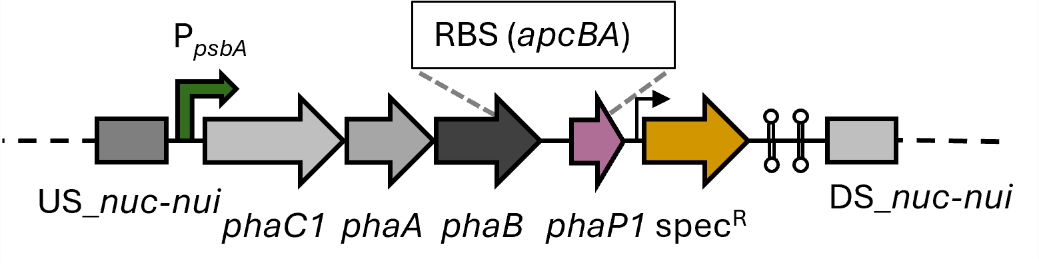


Additional file 9 Fig. S 5: Schematic image of modified PHB in pPF10 for creating recombinant *Nostoc* strain NosPHB3.0; US_*nuc-nui:* Upstream region (1000 bp) of neutral site *nuc-nui*; P*_psbA_*: constitutive promotor; *phaC1*; PHB polymerase; *phaA*: β-ketothiolase*; phaB:* acetoacetyl-CoA reductase; RBS(apcBA): native, intergenic region of *apcBA* with native ribosomal binding site from *Nostoc* sp. 7120; *phaP1*: phasin spec^R^: spectinomycin resistance gene *aad1*, an adenylytransferase; DS_*nuc-nui:* Downstream region (1000 bp) of neutral site *nuc-nui.*

Additional file 10 Table S 5: nucleotide sequence of modified PHB operon in pPF10

| >US_*nuc-nui*: catttgaggtttttttgtgggaaggatctgcacctcctgttacacatgaaatagttttgcagcaaacaggtcacggacaagatgcgccttttaaagtggtagacattgacagcttttttagcagagccactactccccaagactggtatgaggatgaagaaaatgctgtagttgctaaatttcaaaaactgctagaggtaataaaatcgaacttaaaaaacccgcaggtgtatcgactgggtgaggtagaacttgatgtttatgttattggtgaaactccagcaggaaatttagctggtatttctactaaagttgtggaaacttgacctatttactaattatcaactttactctcaatacttgtttgaatattgggggaaacattagacaaaaaatcataacccgtcaaactttctaattcatcaacactgactttataagccctccagtcattatttaattctgggtcgttgggaatatttactgcgataacgcgagtattagcagtaataccttcaagccctgagcctgggctatctagtacgacaacaatcttccaagtggatttgggaactgtcaccttacctttgaggggtttgccaagactaccattaggcccggcaacaatgtaaagctctttaccctgactgactaattctcgacaataatcttctaaatttccccacgtatttctattgttatcgggtgtttggggcatcatgtttgtcatcaggaaagtagccgcattatcttctgttgtcttggtgcggtctgctgaaggtgcaatatgcccccggtcataaccactcccagagtacatagaaggagtcactcgcacccaacccgcaggcaatgttttgtctgggcggaagttatcttgacgctctgcgttccctagccatgaggagttaagctgccaagctacccagttagcagttcccttgctgttgttgtaggagagtgcatattgatttttgaccatcaggtaattatcaggtgt |
| --- |
| >constitutive promotor P*_psbA_*:  TCTAGAGGATCTCAATGAATATTGGTTGACACGGGCGTATAAGACATGTTATACTGTTGAATAACAAGGACGGATCTGATCAAGAGACAGGATGAGGATC |
| PHB operon *phaC1AB:*  ATGGCGACCGGCAAAGGCGCGGCAGCTTCCACGCAGGAAGGCAAGTCCCAACCATTCAAGGTCACGCCGGGGCCATTCGATCCAGCCACATGGCTGGAATGGTCCCGCCAGTGGCAGGGCACTGAAGGCAACGGCCACGCGGCCGCGTCCGGCATTCCGGGCCTGGATGCGCTGGCAGGCGTCAAGATCGCGCCGGCGCAGCTGGGTGATATCCAGCAGCGCTACATGAAGGACTTCTCAGCGCTGTGGCAGGCCATGGCCGAGGGCAAGGCCGAGGCCACCGGTCCGCTGCACGACCGGCGCTTCGCCGGCGACGCATGGCGCACCAACCTCCCATATCGCTTCGCTGCCGCGTTCTACCTGCTCAATGCGCGCGCCTTGACCGAGCTGGCCGATGCCGTCGAGGCCGATGCCAAGACCCGCCAGCGCATCCGCTTCGCGATCTCGCAATGGGTCGATGCGATGTCGCCCGCCAACTTCCTTGCCACCAATCCCGAGGCGCAGCGCCTGCTGATCGAGTCGGGCGGCGAATCGCTGCGTGCCGGCGTGCGCAACATGATGGAAGACCTGACACGCGGCAAGATCTCGCAGACCGACGAGAGCGCGTTTGAGGTCGGCCGCAATGTCGCGGTGACCGAAGGCGCCGTGGTCTTCGAGAACGAGTACTTCCAGCTGTTGCAGTACAAGCCGCTGACCGACAAGGTGCACGCGCGCCCGCTGCTGATGGTGCCGCCGTGCATCAACAAGTACTACATCCTGGACCTGCAGCCGGAGAGCTCGCTGGTGCGCCATGTGGTGGAGCAGGGACATACGGTGTTTCTGGTGTCGTGGCGCAATCCGGACGCCAGCATGGCCGGCAGCACCTGGGACGACTACATCGAGCACGCGGCCATCCGCGCCATCGAAGTCGCGCGCGACATCAGCGGCCAGGACAAGATCAACGTGCTCGGCTTCTGCGTGGGCGGCACCATTGTCTCGACCGCGCTGGCGGTGCTGGCCGCGCGCGGCGAGCACCCGGCCGCCAGCGTCACGCTGCTGACCACGCTGCTGGACTTTGCCGACACGGGCATCCTCGACGTCTTTGTCGACGAGGGCCATGTGCAGTTGCGCGAGGCCACGCTGGGCGGCGGCGCCGGCGCGCCGTGCGCGCTGCTGCGCGGCCTTGAGCTGGCCAATACCTTCTCGTTCTTGCGCCCGAACGACCTGGTGTGGAACTACGTGGTCGACAACTACCTGAAGGGCAACACGCCGGTGCCGTTCGACCTGCTGTTCTGGAACGGCGACGCCACCAACCTGCCGGGGCCGTGGTACTGCTGGTACCTGCGCCACACCTACCTGCAGAACGAGCTCAAGGTACCGGGCAAGCTGACCGTGTGCGGCGTGCCGGTGGACCTGGCCAGCATCGACGTGCCGACCTATATCTACGGCTCGCGCGAAGACCATATCGTGCCGTGGACCGCGGCCTATGCCTCGACCGCGCTGCTGGCGAACAAGCTGCGCTTCGTGCTGGGTGCGTCGGGCCATATCGCCGGTGTGATCAACCCGCCGGCCAAGAACAAGCGCAGCCACTGGACTAACGATGCGCTGCCGGAGTCGCCGCAGCAATGGCTGGCCGGCGCCATCGAGCATCACGGCAGCTGGTGGCCGGACTGGACCGCATGGCTGGCCGGGCAGGCCGGCGCGAAACGCGCCGCGCCCGCCAACTATGGCAATGCGCGCTATCGCGCAATCGAACCCGCGCCTGGGCGATACGTCAAAGCCAAGGCATGACGCTTGCATGAGTGCCGGCGTGCGTCATGCACGGCGCCGGCAGGCCTGCAGGTTCCCTCCCGTTTCCATTGAAAGGACTACACAATGACTGACGTTGTCATCGTATCCGCCGCCCGCACCGCGGTCGGCAAGTTTGGCGGCTCGCTGGCCAAGATCCCGGCACCGGAACTGGGTGCCGTGGTCATCAAGGCCGCGCTGGAGCGCGCCGGCGTCAAGCCGGAGCAGGTGAGCGAAGTCATCATGGGCCAGGTGCTGACCGCCGGTTCGGGCCAGAACCCCGCACGCCAGGCCGCGATCAAGGCCGGCCTGCCGGCGATGGTGCCGGCCATGACCATCAACAAGGTGTGCGGCTCGGGCCTGAAGGCCGTGATGCTGGCCGCCAACGCGATCATGGCGGGCGACGCCGAGATCGTGGTGGCCGGCGGCCAGGAAAACATGAGCGCCGCCCCGCACGTGCTGCCGGGCTCGCGCGATGGTTTCCGCATGGGCGATGCCAAGCTGGTCGACACCATGATCGTCGACGGCCTGTGGGACGTGTACAACCAGTACCACATGGGCATCACCGCCGAGAACGTGGCCAAGGAATACGGCATCACACGCGAGGCGCAGGATGAGTTCGCCGTCGGCTCGCAGAACAAGGCCGAAGCCGCGCAGAAGGCCGGCAAGTTTGACGAAGAGATCGTCCCGGTGCTGATCCCGCAGCGCAAGGGCGACCCGGTGGCCTTCAAGACCGACGAGTTCGTGCGCCAGGGCGCCACGCTGGACAGCATGTCCGGCCTCAAGCCCGCCTTCGACAAGGCCGGCACGGTGACCGCGGCCAACGCCTCGGGCCTGAACGACGGCGCCGCCGCGGTGGTGGTGATGTCGGCGGCCAAGGCCAAGGAACTGGGCCTGACCCCGCTGGCCACGATCAAGAGCTATGCCAACGCCGGTGTCGATCCCAAGGTGATGGGCATGGGCCCGGTGCCGGCCTCCAAGCGCGCCCTGTCGCGCGCCGAGTGGACCCCGCAAGACCTGGACCTGATGGAGATCAACGAGGCCTTTGCCGCGCAGGCGCTGGCGGTGCACCAGCAGATGGGCTGGGACACCTCCAAGGTCAATGTGAACGGCGGCGCCATCGCCATCGGCCACCCGATCGGCGCGTCGGGCTGCCGTATCCTGGTGACGCTGCTGCACGAGATGAAGCGCCGTGACGCGAAGAAGGGCCTGGCCTCGCTGTGCATCGGCGGCGGCATGGGCGTGGCGCTGGCAGTCGAGCGCAAATAAGGAAGGGGTTTTCCGGGGCCGCGCGCGGTTGGCGCGGACCCGGCGACGATAACGAAGCCAATCAAGGAGTGGACATGACTCAGCGCATTGCGTATGTGACCGGCGGCATGGGTGGTATCGGAACCGCCATTTGCCAGCGGCTGGCCAAGGATGGCTTTCGTGTGGTGGCCGGTTGCGGCCCCAACTCGCCGCGCCGCGAAAAGTGGCTGGAGCAGCAGAAGGCCCTGGGCTTCGATTTCATTGCCTCGGAAGGCAATGTGGCTGACTGGGACTCGACCAAGACCGCATTCGACAAGGTCAAGTCCGAGGTCGGCGAGGTTGATGTGCTGATCAACAACGCCGGTATCACCCGCGACGTGGTGTTCCGCAAGATGACCCGCGCCGACTGGGATGCGGTGATCGACACCAACCTGACCTCGCTGTTCAACGTCACCAAGCAGGTGATCGACGGCATGGCCGACCGTGGCTGGGGCCGCATCGTCAACATCTCGTCGGTGAACGGGCAGAAGGGCCAGTTCGGCCAGACCAACTACTCCACCGCCAAGGCCGGCCTGCATGGCTTCACCATGGCACTGGCGCAGGAAGTGGCGACCAAGGGCGTGACCGTCAACACGGTCTCTCCGGGCTATATCGCCACCGACATGGTCAAGGCGATCCGCCAGGACGTGCTCGACAAGATCGTCGCGACGATCCCGGTCAAGCGCCTGGGCCTGCCGGAAGAGATCGCCTCGATCTGCGCCTGGTTGTCGTCGGAGGAGTCCGGTTTCTCGACCGGCGCCGACTTCTCGCTCAACGGCGGCCTGCATATGGGCTGA |
| >RBS(*apcBA*):  ggtgcagctgtttccctgctgaaactggaatattgcaataaggttggaaataaggaactaacaac |
| >*phaP1:*  ATGATCCTCACCCCGGAACAAGTTGCAGCAGCGCAAAAGGCCAACCTCGAAACGCTGTTCGGCCTGACCACCAAGGCGTTTGAAGGCGTCGAAAAGCTCGTCGAGCTGAACCTGCAGGTCGTCAAGACTTCGTTCGCAGAAGGCGTTGACAACGCCAAGAAGGCGCTGTCGGCCAAGGACGCACAGGAACTGCTGGCCATCCAGGCCGCAGCCGTGCAGCCGGTTGCCGAAAAGACCCTGGCCTACACCCGCCACCTGTATGAAATCGCTTCGGAAACCCAGAGCGAGTTCACCAAGGTAGCCGAGGCTCAACTGGCCGAAGGCTCGAAGAACGTGCAAGCGCTGGTCGAGAACCTCGCCAAGAACGCCCCGGCCGGTTCGGAATCGACCGTGGCCATCGTGAAGTCGGCGATCTCCGCTGCCAACAACGCCTACGAGTCGGTGCAGAAGGCGACCAAGCAAGCGGTCGAAATCGCTGAAACCAACTTCCAGGCTGCGGCTACGGCTGCCACCAAGGCTGCCCAGCAAGCCAGCGCCACGGCCCGTACGGCCACGGCAAAGAAGACGACGGCTGCCTGA |
| >*aad1*:  GAGCTCTTGACCGAACGCAGCGGTGGTAACGGCGCAGTGGCGGTTTTCATGGCTTGTTATGACTGTTTTTTTGGGGTACAGTCTATGCCTCGGGCATCCAAGCAGCAAGCGCGTTACGCCGTGGGTCGATGTTTGATGTTATGGAGCAGCAACGATGTTACGCAGCAGGGCAGTCGCCCTAAAACAAAGTTAAACATCATGAGGGAAGCGGTGATCGCCGAAGTATCGACTCAACTATCAGAGGTAGTTGGCGTCATCGAGCGCCATCTCGAACCGACGTTGCTGGCCGTACATTTGTACGGCTCCGCAGTGGATGGCGGCCTGAAGCCACACAGTGATATTGATTTGCTGGTTACGGTGACCGTAAGGCTTGATGAAACAACGCGGCGAGCTTTGATCAACGACCTTTTGGAAACTTCGGCTTCCCCTGGAGAGAGCGAGATTCTCCGCGCTGTAGAAGTCACCATTGTTGTGCACGACGACATCATTCCGTGGCGTTATCCAGCTAAGCGCGAACTGCAATTTGGAGAATGGCAGCGCAATGACATTCTTGCAGGTATCTTCGAGCCAGCCACGATCGACATTGATCTGGCTATCTTGCTGACAAAAGCAAGAGAACATAGCGTTGCCTTGGTAGGTCCAGCGGCGGAGGAACTCTTTGATCCGGTTCCTGAACAGGATCTATTTGAGGCGCTAAATGAAACCTTAACGCTATGGAACTCGCCGCCCGACTGGGCTGGCGATGAGCGAAATGTAGTGCTTACGTTGTCCCGCATTTGGTACAGCGCAGTAACCGGCAAAATCGCGCCGAAGGATGTCGCTGCCGACTGGGCAATGGAGCGCCTGCCGGCCCAGTATCAGCCCGTCATACTTGAAGCTAGACAGGCTTATCTTGGACAAGAAGAAGATCGCTTGGCCTCGCGCGCAGATCAGTTGGAAGAATTTGTCCACTACGTGAAAGGCGAGATCACCAAGGTAGTCGGCAAATAA |
| >t_1_t_2_ terminator:  GCAGAAGCGGTCTGATAAAACAGAATTTGCCTGGCGGCAGTAGCGCGGTGGTCCCACCTGACCCCATGCCGAACTCAGAAGTGAAACGCCGTAGCGCCGATGGTAGTGTGGGGTCTCCCCATGCGAGAGTAGGGAACTGCCAGGCATCAAATAAAACGAAAGGCTCAGTCGAAAGACTGGGCCTTTCGTTTTATCTGTTGTTTGTCGGTGAACGCTCTCCTGAGTAGGACAAATCCGCCGGGAGCGGATTTGAACGTTGCGAAGCAACGGCCCGGAGGGTGGCGGGCAGGACGCCCGCCATAAACTGCCAGGCATCAAATTAAGCAGAAGGCCATCCTGACGGATGGCCTTTTTGCGTTTCTACAAACTC |
| >DS_nuc-nui:  tgttggcgttgcaccactgggatttcccagcagtaaatgcacgctgattgatggggaaagttcagttaatggtggcacttgggattggacaggcgaacacccgacaatcaacgccaccaacgccgctacacccaattttccacaaattcccataaattgacgcacctttagagatttatttcacttcaacacttgtgattgtatttcctgactttcactcagctaggcttcaaattttcaagtctcctgcccgatcaaccctaatttttcctctaaaaaaattagggttgctgacgatcagctacgctcttgcgttacgccatcgctctttcggtgtagcgtaggtcatcgcttacttttcctctctgcgagaggcgggagcgaacgcttgaatttggcggaagggctaaaccaaactcaattgcttcccgtattcctgctccaaacactcccccttgagtctaaagaaacacacacaccacctatcacccaccacccgttcaataccatgcggctgcttgttgtcaaaagcaatgcaatcgccctcctgcgttcatctcagtgtgccatatagtaagaaatagtaagaaaaattgctactttagagtaaaaataatgtattactatgtctaaagtataaaaaatcagtgttgccctgaccccagaaatggtagttttggttcgtgatgctgttgagtcaggagaatatgctagtagcagtgaggtaattcgtgaggcactgcgcgaatggaggcaaaaacggttacttcaattgcaaaatattgaggaactgcgccgtctttggcatgaaggaatggaaagtggtactggacgctttacagatatagaagccatcaagcaagaagcacgctctcgcttaggtcaaacaattcaaaaggatactgaactcaagtgggacgtttaattcgcacagctaaagcagaagaagacttaattgaaatatggatgtacattgcagttgagaacccagaagctgctg |


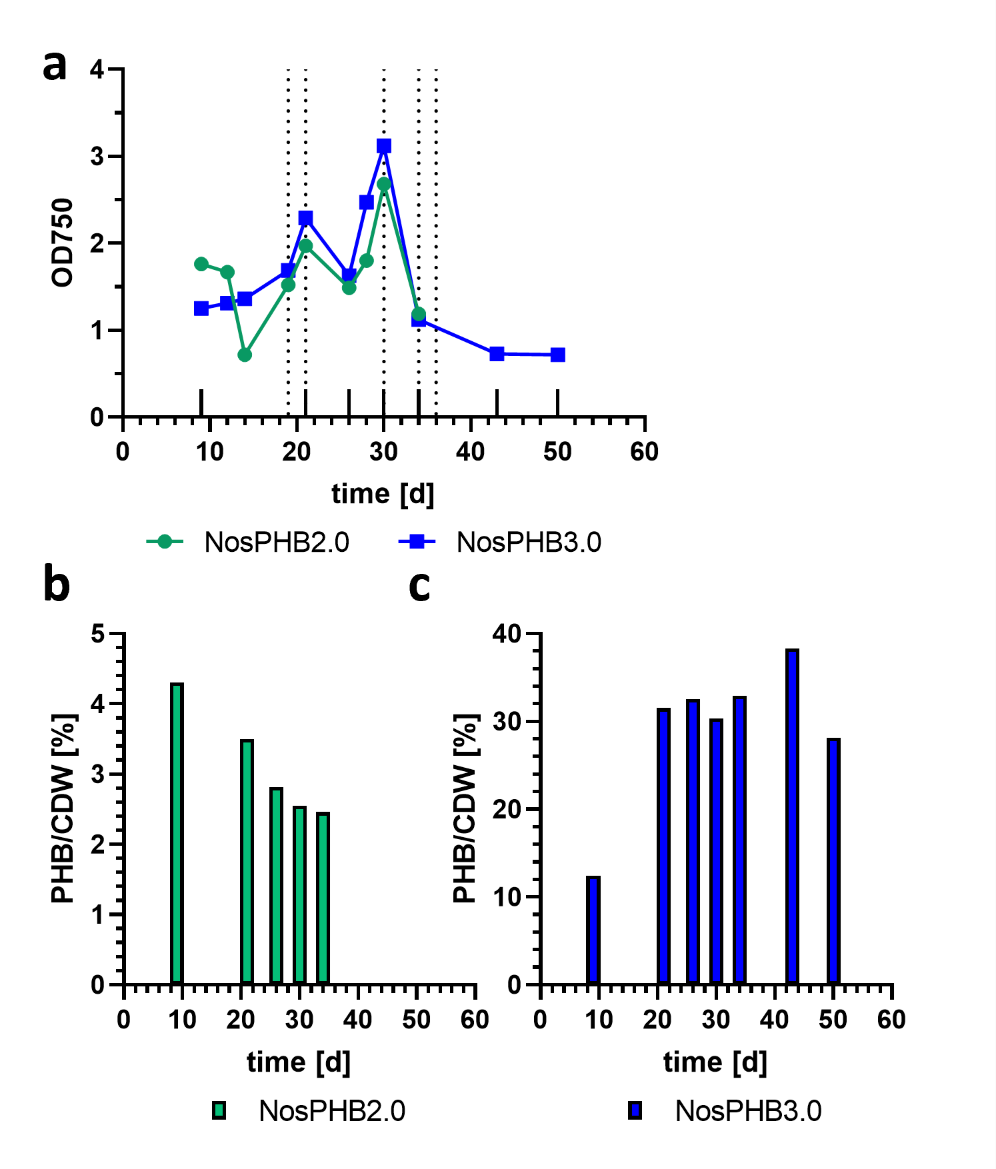


Additional file 11 Fig. S 6: Growth experiment in BG11 medium and PHB quantification of “seed” cultures of NosPHB2.0 and NosPHB3.0 **(a)** Growth curve of NosPHB2.0 (green) and NosPHB3.0 (blue), recorded by measuring the OD_750_. Recording of growth started at day 9. Longer ticks on the x-axis indicate samples taken for PHB quantification. Each point represents one biological replica recorded by measuring the OD_750_. Longer ticks on the x-axis indicate samples taken for PHB quantification. quantification, dashed lines indicate the refilling of BG11 medium to the original volume with appropriate antibiotics. Each point represents one biological experiment. **(b+c)** PHB content of NosPHB2.0 (green, **b**) and NosPHB3.0 (blue, **c**) after the respective days of continuous growth conditions. Each data set represents one biological sample.


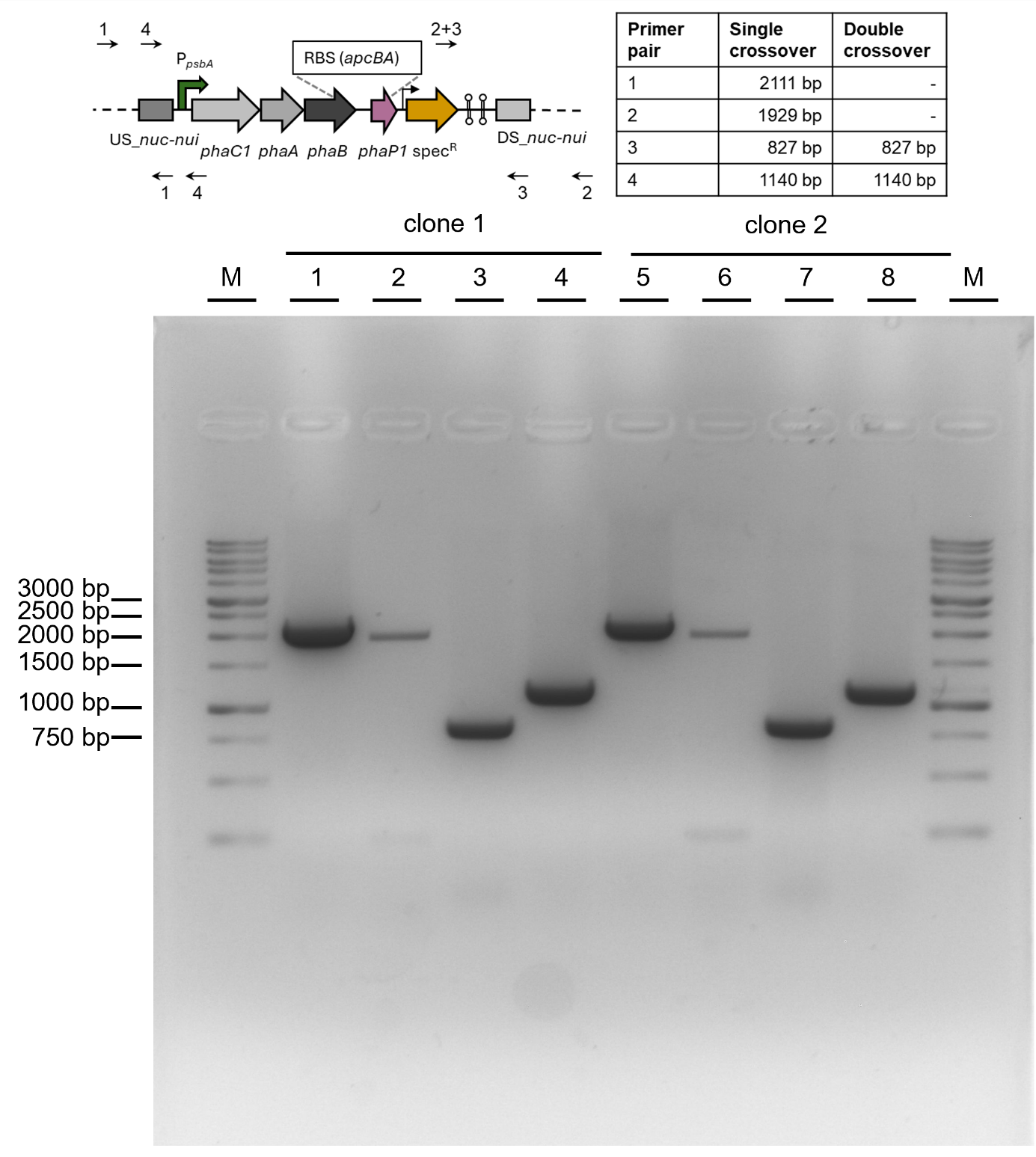


Additional file 12: Fig. S 7: Verification of successful genome integration of PHB operon in recombinant Nostoc strain NosPHB3.0; primer pairs 1-4 were chosen to verify successful integration of improved PHB operon in recombinant *Nostoc* strain M: marker; line 1-4; clone 1 with primer pair 1-4 respectively; line 5-8; clone 4 with primer pair 1-4 respectively. Double crossover event was not achieved in clone 1 and 2 of NosPHB3.0.


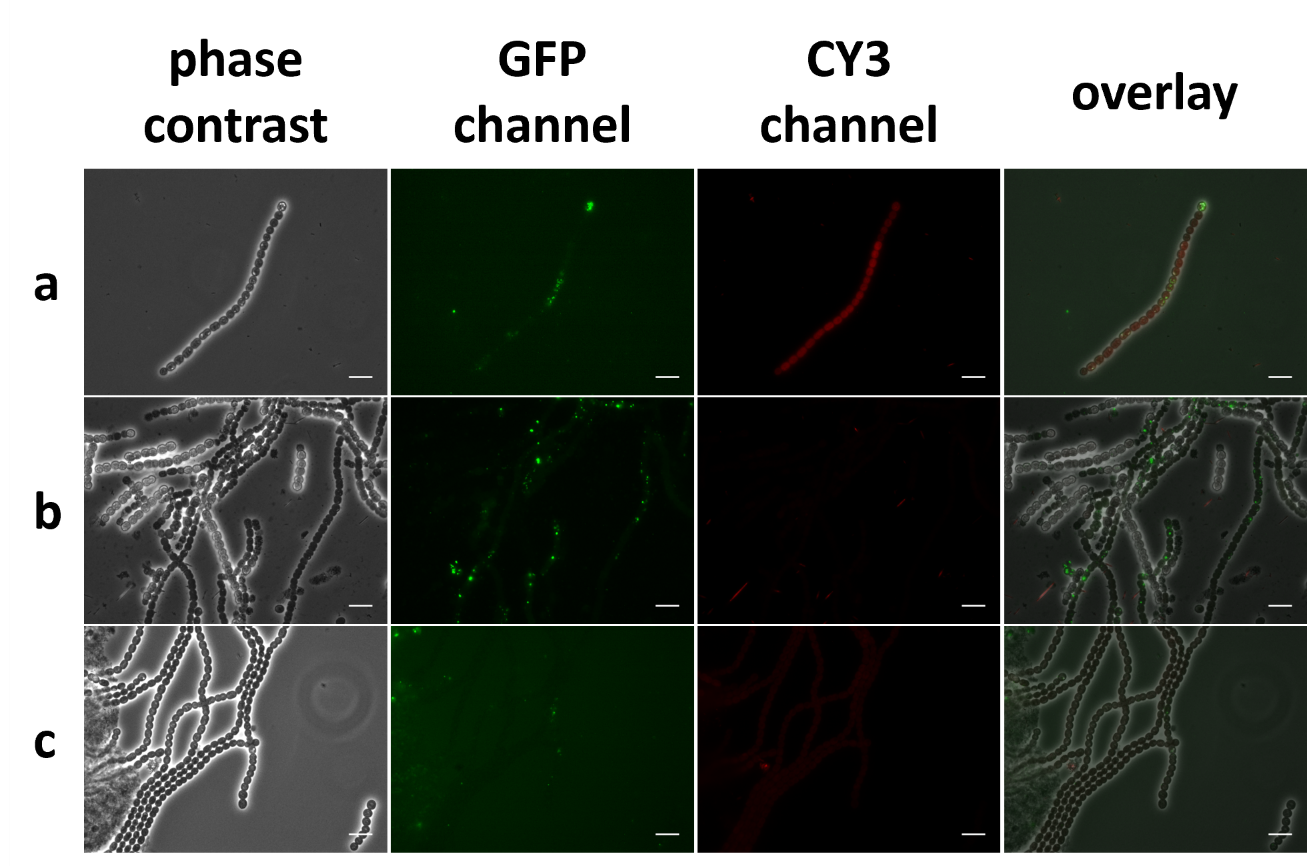


Additional file 13: Fig. S 8: Microscopic images of NosPHB2.0 after 3 days of continuous growth condition **(a)** NS: 5 µmol m^-2^s^-1^, 0 rpm; **(b)** S: 50-60 µmol m^-2^s^-1^, 120 rpm; **(c)** DS: 20 µmol m^-2^s^-1^, 120 rpm, PHB granules are visualized by BODIPY staining and detected using the GFP channel PHB granules are visualized with BODIPY staining (GFP channel), autofluorescence (CY3 channel), overlay of phase contrast, GFP and CY3 channel, heterocyst formation was indicated with white arrows,scale bar = 10 µm.


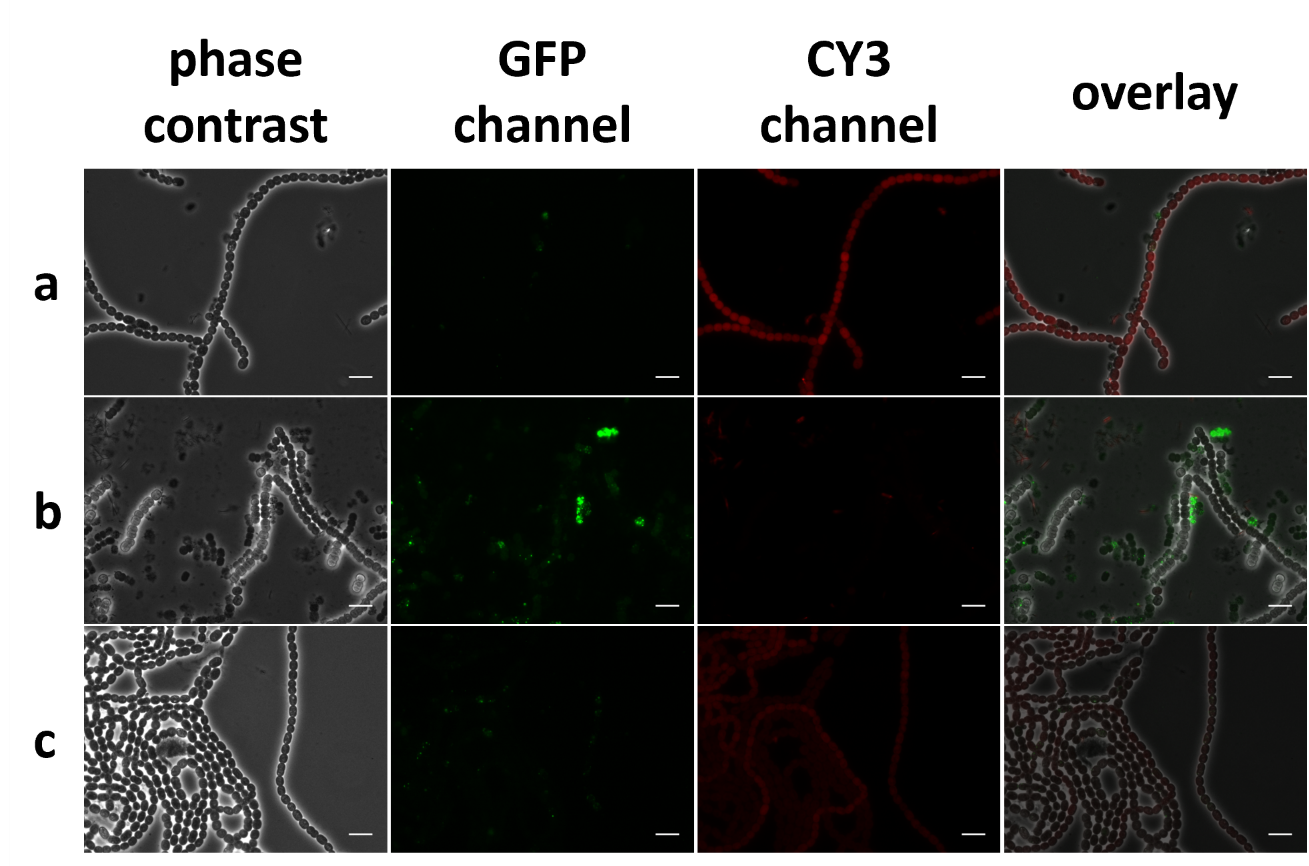


Additional file 14: Fig. S 9: Microscopic images of NosPHB2.0 after 7 days of continuous growth condition **(a)** NS: 5 µmol m^-2^s^-1^, 0 rpm; **(b)** S: 50-60 µmol m^-2^s^-1^, 120 rpm; **(c)** DS: 20 µmol m^-2^s^-1^, 120 rpm, PHB granules are visualized by BODIPY staining and detected using the GFP channel PHB granules are visualized with BODIPY staining (GFP channel), autofluorescence (CY3 channel), overlay of phase contrast, GFP and CY3 channel, heterocyst formation was indicated with white arrows, scale bar = 10 µm.


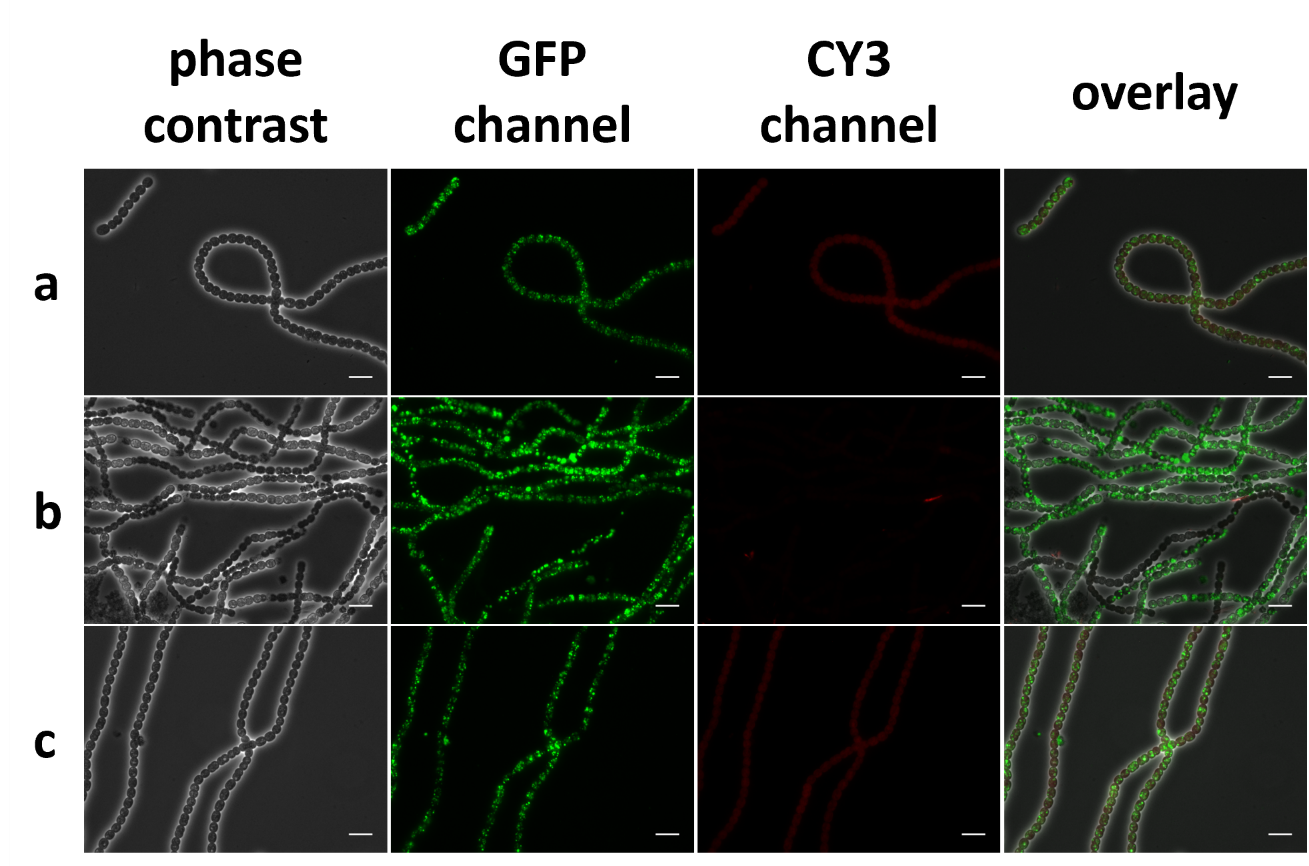


Additional file 15: Fig. S 10: Microscopic images of NosPHB3.0 after 3 days of continuous growth condition **(a)** NS: 5 µmol m^-2^s^-1^, 0 rpm; **(b)** S: 50-60 µmol m^-2^s^-1^, 120 rpm; **(c)** DS: 20 µmol m^-2^s^-1^, 120 rpm, PHB granules are visualized by BODIPY staining and detected using the GFP channel PHB granules are visualized with BODIPY staining (GFP channel), autofluorescence (CY3 channel), overlay of phase contrast, GFP and CY3 channel, heterocyst formation was indicated with white arrows, scale bar = 10 µm.


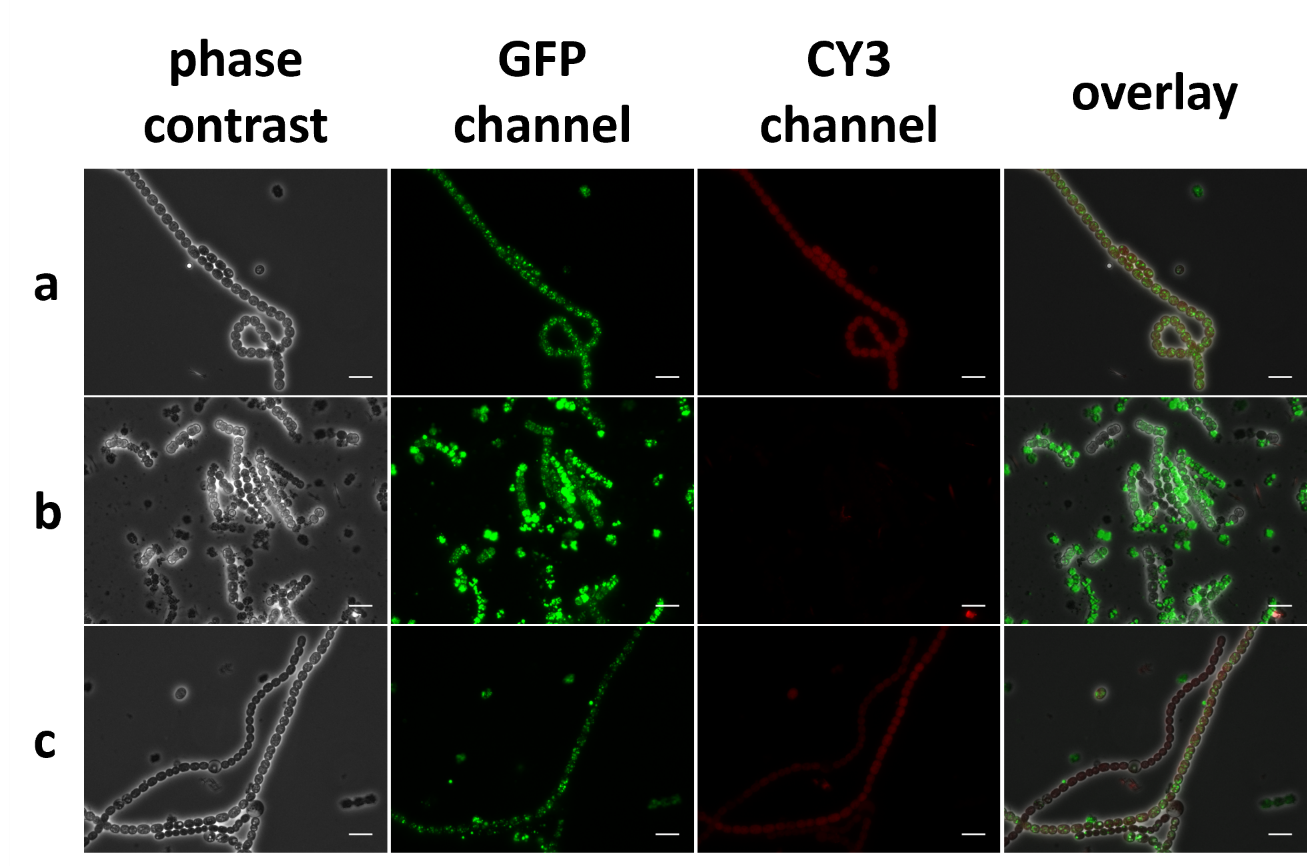


Additional file 16: Fig. S 11: Microscopic images of NosPHB3.0 after 7 days of continuous growth condition **(a)** NS: 5 µmol m^-2^s^-1^, 0 rpm; **(b)** S: 50-60 µmol m^-2^s^-1^, 120 rpm; **(c)** DS: 20 µmol m^-2^s^-1^, 120 rpm, PHB granules are visualized by BODIPY staining and detected using the GFP channel PHB granules are visualized with BODIPY staining (GFP channel), autofluorescence (CY3 channel), overlay of phase contrast, GFP and CY3 channel, heterocyst formation was indicated with white arrows, scale bar = 10 µm.

**References**

1. Datta N, Hedges RW, Shaw EJ, Sykes RB, Richmond MH. Properties of an R Factor from Pseudomonas aeruginosa. Journal of Bacteriology. 1971 Dec;108(3):1244–9.

2. Elhai J, Vepritskiy A, Muro-Pastor AM, Flores E, Wolk CP. Reduction of conjugal transfer efficiency by three restriction activities of Anabaena sp. strain PCC 7120. Journal of Bacteriology. 1997 Mar;179(6):1998–2005.

3. Cai YP, Wolk CP. Use of a conditionally lethal gene in Anabaena sp. strain PCC 7120 to select for double recombinants and to entrap insertion sequences. Journal of Bacteriology. 1990 Jun;172(6):3138–45.

4. Black TA, Wolk CP. Analysis of a Het- mutation in Anabaena sp. strain PCC 7120 implicates a secondary metabolite in the regulation of heterocyst spacing. Journal of Bacteriology. 1994 Apr;176(8):2282–92.
